# Emerging evidence and treatment paradigm of non-small cell lung cancer

**DOI:** 10.1186/s13045-023-01436-2

**Published:** 2023-04-17

**Authors:** Si-Yang Maggie Liu, Mei-Mei Zheng, Yi Pan, Si-Yang Liu, Yangqiu Li, Yi-Long Wu

**Affiliations:** 1 Department of Hematology, The First Affiliated Hospital, Jinan University, Guangzhou, 510632 China; 2 Guangdong Lung Cancer Institute, Guangdong Provincial People’s Hospital (Guangdong Academy of Medical Sciences), Southern Medical University, 106 Zhongshan Er Road, Guangzhou, 510080 China; 3 Institute of Hematology, School of Medicine, Key Laboratory for Regenerative Medicine of Ministry of Education, Jinan University, Guangzhou, 510632 China; 4 Guangdong Lung Cancer Institute, Guangdong Provincial Key Laboratory of Translational Medicine in Lung Cancer, Guangdong Provincial People’s Hospital (Guangdong Academy of Medical Sciences), Southern Medical University, Guangzhou, 510080 China

## Abstract

Research on biomarker-driven therapy and immune check-point blockade in non-small cell lung cancer (NSCLC) is rapidly evolving. The width and depth of clinical trials have also dramatically improved in an unprecedented speed. The personalized treatment paradigm evolved every year. In this review, we summarize the promising agents that have shifted the treatment paradigm for NSCLC patients across all stages, including targeted therapy and immunotherapy using checkpoint inhibitors. Based on recent evidence, we propose treatment algorithms for NSCLC and propose several unsolved clinical issues, which are being explored in ongoing clinical trials. The results of these trials are likely to impact future clinical practice.

## Background

Lung cancer is the leading cause of cancer-related deaths worldwide [1]. Previously, the only systemic antitumor therapy available was chemotherapy, which had modest efficacy and high toxicity. In recent years, small molecular tyrosine kinase inhibitors (TKIs) have emerged as promising treatment options [2] that lead to tumor regression. Non-small cell lung cancer (NSCLC) patients with driver gene mutations who are treated with TKIs have favorable survival outcomes and low toxicity [3, 4]. The development of epidermal growth factor receptor (EGFR)-TKIs has revolutionized the personalized treatment paradigm for NSCLC patients with *EGFR* mutations [5]. The median overall survival (OS) of such patients with advanced disease increased from < 10 months to almost 40 months [6, 7]. Immune checkpoint inhibitors (ICIs), such as programmed death 1 (PD-1) or programmed death ligand 1 (PD-L1) inhibitors, destroy tumor cells via the PD-L1/PD-1 pathway and reactivate effector T cells [8]. The development of ICIs is also an important milestone in lung cancer treatment [9]. In patients without driver gene mutations, PD-L1/PD-1 inhibitors produce a durable clinical response and prolong long-term OS [10–12]. After all these years, EGFR-TKIs targeting the first druggable oncogene has evolved from first- to third-generation; furthermore, regulatory approvals have also been granted for agents directed against at least 9 another molecular targets. ICI monotherapy and combination therapy with chemotherapy have been approved for clinical use [13]. Here, we discuss the promising agents that have shifted the treatment paradigm for NSCLC patients across all stages, including targeted therapy and immunotherapy. In particular, studies conducted in China are also discussed to improve the generalizability of our findings and provide an important reference for studies conducted in other countries. Additionally, we have highlighted several clinical issues that should be explored by future studies.

## Early-stage NSCLC

For the past 20 years, neoadjuvant or adjuvant chemotherapy has been recommended for patients with resectable NSCLC. However, the 5-year OS has improved by only 5% [14]. Novel adjuvant strategies are needed to optimize clinical outcomes after complete surgical resection in patients with early-stage NSCLC. A long-term survival benefit has been achieved with the use of EGFR-TKIs and ICIs in advanced NSCLC patients [6, 7, 11]. The treatment paradigm of such patients has changed significantly [15]. However, a few unresolved issues remain, including whether this success will be repeated in perioperative management of resectable NSCLC and how the treatment will be modified in the future. In this section, we review the results of important phase III clinical trials and propose unresolved clinical issues.

### Emerging evidence with clinical implications

#### Adjuvant therapy with EGFR-TKIs

In the phase III ADAURA study, the third-generation EGFR*-*TKI osimertinib showed a clinically meaningful improvement in disease-free survival (DFS) in patients with resected *EGFR*-mutant NSCLC [16]. The risks of disease recurrence and death were reduced by 77% in patients with stage II–IIIA disease and by 73% in those with stage IB–IIIA disease. The survival benefit was greater in patients with more advanced disease, based on the eighth edition of the TNM classification (stage IB, hazard ratio [HR] = 0.44; stage II, HR = 0.33; stage IIIA, HR = 0.22) [17]. A DFS benefit was observed in patients treated with (HR = 0.29) or without (HR = 0.36) adjuvant chemotherapy, regardless of disease stage [18]. Despite the lack of data on OS, osimertinib was approved by the Food and Drug Administration (FDA) and other regulatory authorities as adjuvant therapy for patients with *EGFR* exon 19 deletion or the exon 21 L858R mutation [19].

Three phase III studies have shown conflicting findings regarding the use of first-generation EGFR-TKIs compared with chemotherapy as adjuvant treatment for resected *EGFR*-mutant NSCLC. The first one is the CTONG1104 study. A randomized phase III trial showed that adjuvant gefitinib, a first-generation EGFR-TKI, significantly prolonged the median DFS compared with chemotherapy (30.8 vs. 20.8 months) in patients with stage II–IIIA NSCLC harboring *EGFR* mutations [20]. The risk of recurrence or death was reduced by 44% (p = 0.001). However, the median OS was not significantly different between the gefitinib and chemotherapy groups over a median follow-up duration of 80 months (75.5 vs. 62.8 months; HR = 0.92; *p * = 0.674) [21]. The EVIDENCE study compared adjuvant icotinib with chemotherapy for patients with stage II–IIIA *EGFR*-mutant NSCLC. After a median follow-up duration of 24.9 months, the median DFS was longer in the icotinib group than in the chemotherapy group (47.0 vs. 22.1 months; HR = 0.36; *p * < 0.0001) [22]. Despite the lack of data on OS, the National Medical Products Administration approved icotinib as adjuvant treatment for patients with *EGFR* mutations after complete resection. The IMPACT study had a similar design to the CTONG1104 trial, which compared adjuvant gefitinib with chemotherapy for patients with resected stage II–IIIA NSCLC harboring *EGFR* mutations [23]. Although adjuvant gefitinib prevented early relapse, it did not prolong DFS or OS. The median DFSs were 35.9 and 25.1 months in the gefitinib and chemotherapy groups, respectively (HR = 0.92; *p * = 0.63), whereas the median OS was not reached (HR = 1.03). Because the Kaplan–Meier curves overlapped at 3–4 years after surgery, adjuvant therapy with gefitinib was not approved for clinical use.

#### Adjuvant or neoadjuvant therapy with PD-1/PD-L1 inhibitors

IMpower010 was the first phase III trial to compare adjuvant immunotherapy with standard therapy for resected stage IB–IIIA NSCLC [24]. After adjuvant chemotherapy, atezolizumab group was associated with a significantly longer DFS compared with supportive care group. The risks of recurrence and death were reduced after atezolizumab treatment by 34% in stage II–IIIA NSCLC patients with ≥ 1% PD-L1 expression and by 21% in all stage II–IIIA NSCLC patients. The effect of atezolizumab on OS over a median follow-up duration of 32 months was unclear. On October 15, 2021, the FDA approved adjuvant atezolizumab following resection and platinum-based chemotherapy for patients with stage II–IIIA NSCLC and ≥ 1% PD-L1 expression in tumor cells; furthermore, the Ventana PD-L1 (SP263) assay was approved as a companion diagnostic device [25]. The IMpower010 study presented 46-month follow-up data for OS at the 2022 World Conference of Lung Cancer. Although the median OS was not reached, there was a trend toward prolonged OS after atezolizumab treatment in stage II–IIIA patients with ≥ 1% PD-L1 expression in tumor cells (HR = 0.71; 95% confidence interval [CI] = 0.49–1.03). A significant OS advantage was observed in patients with ≥ 50% PD-L1 expression in tumor cells (HR = 0.43; 95% CI = 0.24–0.78) [26].

KEYNOTE-091, the second clinical trial of adjuvant immunotherapy [27], enrolled patients with completely resected stage IB–IIIA NSCLC treated with pembrolizumab or placebo every 3 weeks for up to 18 cycles. Adjuvant chemotherapy was not mandatory for all patients and was recommended according to the local guidelines. The second interim analysis of KEYNOTE-091 was performed after a median follow-up duration of 35.6 months. The median DFS was longer in the pembrolizumab group compared with the placebo group (53.6 vs. 42.0 months; HR = 0.76; *p* = 0.0014). In patients with ≥ 50% PD-L1 expression in tumor cells, pembrolizumab did not prolong the DFS compared with the placebo (HR = 0.82; *p* = 0.14). In patients who received adjuvant chemotherapy, pembrolizumab prolonged the DFS by almost 2 years compared with the placebo (58.7 vs. 34.9 months; HR = 0.73; 95% CI = 0.60–0.89). On January 26, 2023, the FDA approved pembrolizumab as adjuvant treatment following resection and platinum-based chemotherapy for patients with stage IB–IIIA NSCLC [28].

Neoadjuvant therapies have several advantages, including reduction of tumor burden, which allows complete tumor resection, and evaluation of the pathological treatment response. CheckMate816 was the first phase III study that found favorable outcomes of neoadjuvant immunotherapy in patients with resectable stage IB–IIIA NSCLC [29]. The primary endpoint was pathological complete response (pCR) and event-free survival (EFS). Neoadjuvant nivolumab plus chemotherapy, compared with chemotherapy alone, significantly improved the pCR rate (24.0% vs. 2.2%; *p * < 0.0001) and prolonged the EFS by 11 months (31.6 vs. 20.8 months; HR = 0.63; *p * = 0.0052) after a median follow-up duration of 29.5 months. Most subgroups benefited from nivolumab plus chemotherapy compared with chemotherapy alone, and the interim analysis showed a favorable trend for OS (HR = 0.57; *p * = 0.0079). Based on these results, neoadjuvant therapy with nivolumab plus chemotherapy was approved by the FDA on March 4, 2022, and by the National Medical Products Administration in January 2023 for patients with resectable NSCLC [30].

**Fig. 1:**
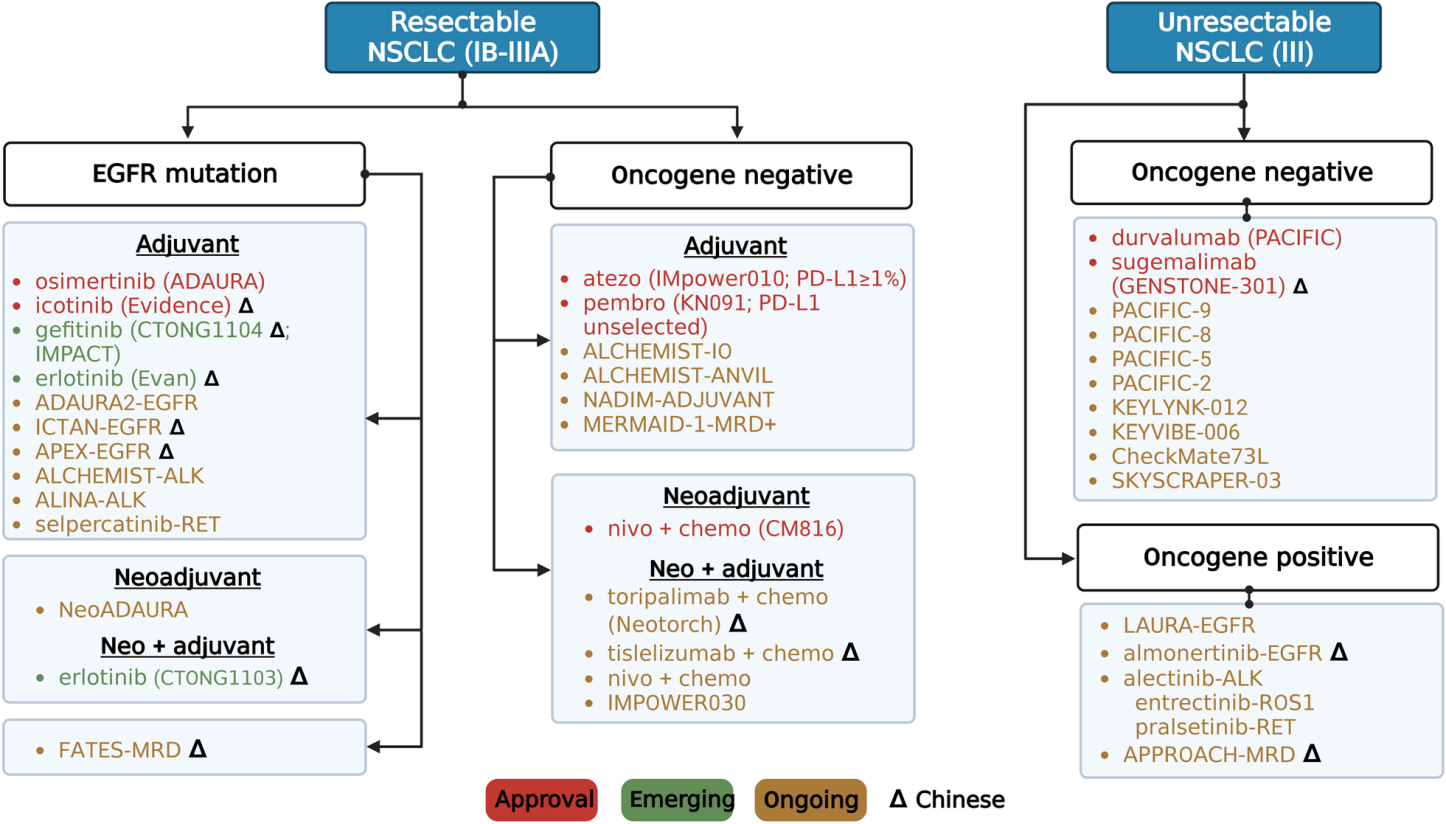
Treatment algorithm for resectable and unresectable NSCLC. Atezo, atezolizumab; nivo, nivolumab; pembro, pembrolizumab; durva, durvalumab; chemo, chemotherapy; CM816, CheckMate816; KN091, KEYNOTE-091

We summarize the shift in the treatment paradigm for early-stage NSCLC based on recently approved agents. We also present select ongoing trials with promising clinical implication in the next 5 years (Fig. 1, Table 1).

**Table 1:** Ongoing trials for resectable earl-stage NSCLC patients that may change clinical practice in 5 years

Agents	Agent type	Control arm	Identification	status	NCT/Name	Sample size	Primary endpoint
*Neoadjuvant + Adjuvant*							
Neoad: Nivolumab + chemo Adj: Nivolumab	PD-1 inhibitor	Placebo + chemo	Stage II–IIIB (N2)	Phase III	NCT04025879	452	EFS
Neoad: Atezolizumab + chemo *4C Adj: Atezolizumab*13C	PD-1 inhibitor	Placebo + chemo	Stage II–IIIB (N2)	Phase III	NCT03456063 (IMpower030)	453	EFS
Neoad: Toripalimab + chemo*3C Adj: Toripalimab + chemo*1C, Toripalimab*13C	PD-1 inhibitor	Placebo + chemo	Stage II–IIIB (N2)	Phase III	NCT04158440	406	MPR rate in stage III population; EFS in stage III population; MPR rate in stage II–III population;
Neoad: Tislelizumab + chemo Adj: Tislelizumab	PD-1 inhibitor	Placebo + chemo	Stage II–IIIA	Phase III	NCT04379635	453	MPR; EFS
*Neoadjuvant*							
Arm1: Osimertinib + chemo Arm2: Osimertinib	EGFR-TKI	Placebo + chemo	Stage II–IIIB N2	Phase III	NCT04351555 (NeoADAURA)	328	MPR
*Adjuvant*							
Arm1: Pembrolizumab + chemo*4C, pembrolizumab; Arm2: chemo*4C, pembrolizumab	PD-1 inhibitor	Chemo	Stage II–IIIB (N2)	Phase III	NCT04267848 (ALCHEMIST)	1210	DFS
Nivolumab + chemo*4C, nivolumab	PD-1 inhibitor	Chemo	Stage IB–IIIA	Phase III	NCT04564157 (NADIM-ADJUVANT)	210	DFS
Nivolumab + chemo	PD-1 inhibitor	Chemo + observation	Stage IB–IIIA	Phase III	NCT02595944 (ALCHEMIST-ANVIL)	903	DFS; OS
Durvalumab	PD-1 inhibitor	Placebo	Stage IB–IIIA	Phase III	NCT02273375	1415	DFS
Durvalumab + chemo	PD-1 inhibitor	Placebo + chemo	stage II–III NSCLC with MRD+	Phase III	NCT04385368 (MERMAID-1)	89	DFS
Osimertinib	EGFR-TKI	Placebo	Stage IA2–IA3	Phase III	NCT05120349 (ADAURA2)	380	DFS in high-risk stratum
Arm1: 6-month icotinib; Arm2: 12-month icotinib;	EGFR-TKI	Chemo	Stage II–IIIA	Phase III	NCT01996098 (ICTAN)	318	DFS
Arm1: Almonertinib + Chemo; Arm2: Almonertinib	EGFR-TKI	Chemo	Stage II–IIIA	Phase III	NCT04762459 (APEX)	606	DFS
Almonertinib	EGFR-TKI	Placebo	Stage II–IIIB (N2)	Phase III	NCT04687241	192	DFS
Gefitinib + chemo*4C, gefitinib	EGFR-TKI	Chemo	Stage II–IIIB (N2)	Phase III	NCT03381066	225	DFS
Alectinib	ALK-TKI	Chemo	Stage IB–IIIA	Phase III	NCT03456076 (ALINA)	257	DFS
Crizotinib	ALK-TKI	Observation	Stage IB–IIIA	Phase III	NCT02201992 (ALCHEMIST)	168	OS
Ensartinib	ALK-TKI	Placebo	Stage II–IIIB (N2)	Phase III	NCT05341583	202	DFS
Selpercatinib	RET-TKI	Placebo	Stage IB–IIIA	Phase III	NCT04819100	170	EFS

*Chemo chemotherapy, *DFS* disease-free survival, *EFS* event-free survival, *MPR* major pathological response, *OS* overall survival, *pCR* pathological complete response, *TKI* tyrosine kinase inhibitor, *C* cycles

#### Unresolved clinical issues

Prospective phase III studies have reported promising results. However, certain clinical issues remain unresolved. First, it is unclear whether adjuvant chemotherapy is necessary for all patients. Most trials of adjuvant treatment, such as CTONG1104 and EVIDENCE, have found beneficial effects of treatment with EGFR-TKIs plus chemotherapy in patients with resected NSCLC [21, 22]. However, the ADAURA study concluded that adjuvant chemotherapy should be administered on a case-by-case basis according to the decision of the patients and their physicians. Osimertinib was associated with prolonged survival compared with the placebo [16], with a lower HR (0.29 vs. 0.39), suggesting beneficial effects of chemotherapy [18]. To resolve this issue, future studies should compare investigational agents plus chemotherapy with investigational agents alone in a large number of patients to detect any differences [31]. Most ongoing clinical trials are using chemotherapy or chemotherapy plus placebo as the control arm. The ongoing APEX study is comparing the efficacy and safety of almonertinib plus chemotherapy, almonertinib alone, and chemotherapy alone for resected *EGFR*-mutant NSCLC [32].

Second, the effects of neoadjuvant therapy on pathological responses are unclear. Immunotherapy has a unique antitumor mechanism of action. When the checkpoint pathway is blocked, tumor-infiltrating lymphocytes and macrophages enter the tumor nest, leading to tumor progression based on RECIST assessment [33] and a major pathological response (MPR) or pCR. Thus, the pathological response is an early therapeutic indicator for neoadjuvant therapy after complete resection and guides treatment decisions. A previous study found that homologous recombination deficiency was associated with the neoadjuvant immunotherapy response in lung cancer [34]. Improvements in MPR and pCR rates were associated with longer EFS and DFS [35–37]. However, it is unclear whether the MPR or pCR rate indicates a survival benefit. Although several phase III neoadjuvant trials used MPR as a primary endpoint, further studies are needed to confirm their results.

Third, it is unclear whether the molecular residual disease (MRD) status determined by liquid biopsy can guide the selection of personalized adjuvant therapy. The MRD status can be determined by sequencing circulating tumor DNA in peripheral blood samples to predict disease recurrence and distant metastasis [38]. The baseline MRD status within 1 month after surgery can be used to stratify patients into high- and low-risk groups [39]. Furthermore, longitudinal MRD surveillance can identify patients with potential to be cured by resection, resulting in a negative predictive value of 96.8% [40]. Additionally, the positive status of MRD can predict disease recurrence 3–5 months earlier than can imaging [41]. Dynamic MRD monitoring after surgery provides treatment guidance regarding the risk of disease recurrence or metastasis. Previous studies have shown that consolidation treatment with ICIs improved the clinical outcomes of patients with MRD [42]. Patients with *EGFR*-mutant advanced disease with MRD negative status after local treatment or tumor burden on imaging may undergo an EGFR-TKI “drug holiday” [43]. However, further studies are needed to determine whether personalized adjuvant therapy can be selected based on the MRD status. Multiple clinical trials with novel designs, such as FATES/CTONG2105, have reported promising results [44]. Accumulation of further evidence may affect the use of adjuvant therapy for early-stage NSCLC.

Fourth, it is also unclear whether adjuvant targeted therapy against rare genetic variants is effective. Rare genetic variants, such as anaplastic lymphoma kinase (*ALK*) and c-ros oncogene 1 (*ROS1*) fusion, have low prevalence and druggability [45]. Chemotherapy and immunotherapy with ICIs have moderate efficacy but high toxicity [46, 47]. Treatment with effective TKIs has changed the treatment paradigm for patients with advanced NSCLC harboring rare genetic variants [48–50]. Tables 1 and 2 summarize the ongoing prospective clinical trials of targeted therapy in patients with rare genetic variants who have undergone resection. The ALINA study, which will compare adjuvant alectinib with adjuvant chemotherapy in patients with *ALK* fusion genes, has completed patient enrollment [51]. Its results may impact future clinical treatment of patients.

**Table 2:** Ongoing trials for unresectable stage III NSCLC patients that may change clinical practice in 5 years

Agents	Agent type	Control arm	Status	Chemo-radiotherapy	NCT/Name	Sample size	Primary endpoint
Arm1:TQB2450 + Anlotinib Arm2: TQB2450	PD-L1 inhibitor	Placebo	Phase III	cCRT/sCRT	NCT04325763	315	PFS
Arm1: Durvalumab + Oleclumab; Arm2: Durvalumab + Monalizumab	PD-L1 inhibitor; CD73 inhibitor; NKG2A inhibitor	Durvalumab + Placebo	Phase III	cCRT	NCT05221840 (PACIFIC-9)	999	PFS
Durvalumab + Domvanalimab	PD-L1 inhibitor; TIGIT inhibitor	Durvalumab + Placebo	Phase III	cCRT	NCT05211895 (PACIFIC-8)	860	PFS
Durvalumab	PD-L1 inhibitor	Placebo	Phase III	cCRT/sCRT	NCT03706690 (PACIFIC-5)	407	PFS
Durvalumab + cCRT → durvalumab	PD-L1 inhibitor	Placebo	Phase III	cCRT	NCT03519971 (PACIFIC-2)	328	PFS
Arm1: Pembrolizumab + cCRT → pembrolizumab; Arm2:Pembrolizumab + cCRT → pembrolizumab + Olaparib	PD-1 inhibitor; PARP inhibitor	Durvalumab	Phase III	cCRT	NCT04380636 (KEYLYNK-012)	870	PFS; OS
Pembrolizumab + vibostolimab + cCRT → pembrolizumab + vibostolimab;	PD-1 inhibitor; TIGIT inhibitor	Durvalumab	Phase III	cCRT	NCT05298423 (KEYVIBE-006)	784	PFS; OS; PFS + OS (PD-L1 ≥ 1%);
Arm1: Nivolumab + cCRT → nivolumab + ipilimumab Arm2: Nivolumab + cCRT → nivolumab	PD-1 inhibitor; CTLA-4 inhibitor	Durvalumab	Phase III	cCRT	NCT04026412 (CheckMate73L)	888	PFS
Atezolizumab + tiragolumab	PD-L1 inhibitor; TIGIT inhibitor	Durvalumab	Phase III	cCRT	NCT04513925 (SKYSCRAPER-03)	800	PFS
Osimertinib	EGFR-TKI	Placebo	Phase III	cCRT/sCRT	NCT03521154 (LAURA)	216	PFS
Almonertinib	EGFR-TKI	Placebo	Phase III	cCRT/sCRT	NCT04951635	150	PFS
Arm1: Alectinib Arm2: Entrectinib Arm3: Pralsetinib	ALK-TKI; ROS1-TKI; RET-TKI	Durvalumab	Phase I-III	cCRT/sCRT	NCT05170204	320	PFS
MRD+: Durvalumab+ Chemotherapy; MRD−: Durvalumab	PD-L1 inhibitor	–	Phase II	cCRT	NCT04585490	48	The change of ctDNA (MRD+) due to the addition of chemotherapy

*PFS* progression-free survival; *cCRT/sCRT* concurrent/sequential chemotherapy and radiotherapy; *OS* overall survival; *TKI* tyrosine kinase inhibitor; *MRD* molecular residual disease; *ctDNA* circulating tumor DNA

## Localized NSCLC

Patients with stage III NSCLC are a highly heterogenous population. Approximately one-third of these patients survive for 5 years, and most have N2 disease and metastasis [52]. These patients have unresectable, resectable, or potentially resectable tumors [53]. In clinical practice, stage III patients with unresectable disease are treated with definitive chemoradiotherapy. We present emerging evidence with clinical implications, summarize the ongoing clinical trials that may change clinical practice in the next 5 years, and highlight the unresolved clinical issues to be addressed.

### Emerging evidence with clinical implications

The PACIFIC trial was the first to impact the clinical treatment of stage III NSCLC patients with unresectable disease. This phase III study compared consolidation therapy with durvalumab and placebo in patients with stage III NSCLC after concurrent chemoradiotherapy [54]. Based on the initial results, on February 16, 2018, the FDA approved durvalumab for patients with unresectable stage III NSCLC following concurrent platinum-based chemotherapy and radiation therapy [55]. The detailed results, published 5 years after the last patient was randomized [12], showed median progression-free survival (PFS) times of 16.9 and 5.6 months in the durvalumab and placebo groups, respectively (HR = 0.55; 95% CI = 0.45–0.68). The 5-year PFS rates were 33.1% and 19.0% in the durvalumab and placebo groups, respectively, indicating that one-third of patients receiving durvalumab were disease-free in 5 years. The median OS times were 47.5 and 29.1 months, and the 5-year OS rates were 42.9% and 33.4%, in the durvalumab and placebo groups, respectively (HR = 0.72; 95% CI = 0.59–0.89). These results have significant implications for unresectable stage III patients. Consistent with previous reports, OS and PFS benefits with durvalumab compared with placebo were observed in all prespecified subgroups. However, given the small sample size, it is uncertain whether durvalumab is associated with a survival benefit in patients with *EGFR* mutations or *ALK* fusion.

Gemstone-301 is a phase III study that compared PFS between sugemalimab and placebo after concurrent or sequential chemoradiotherapy in patients with locally advanced, unresectable, stage III NSCLC in China. Patients were excluded if they had sensitive *EGFR*, *ALK*, or *ROS1* gene alterations. According to the interim results, consolidation therapy with sugemalimab resulted in a statistically and clinically significant prolongation of the median PFS (9.0 vs. 5.8 months) and reduction of recurrence risk by 36% compared with placebo [56] in all patients as well as in the predefined subgroups. Based on these results, on June 6, 2022, sugemalimab was approved in China for the treatment of unresectable stage III NSCLC without progression after concurrent or sequential chemoradiotherapy. Subsequent results of Gemstone-301 showed median PFS times after sugemalimab and placebo treatment of 10.5 and 6.2 months (HR = 0.65), respectively, for both sequential and concurrent chemoradiotherapy; 8.1 and 4.1 months (HR = 0.57), respectively, for sequential chemoradiotherapy; and 15.7 and 8.3 months (HR = 0.71), respectively, for concurrent chemoradiotherapy [57]. The OS in the sugemalimab and placebo groups was inconclusive (not reached vs. 25.9 months) after median follow-up durations of 27.1 and 23.5 months, respectively.

After the approval of durvalumab for stage III disease, the PACIFIC-R study was initiated as an international retrospective trial to assess its real-world effectiveness. Patients received consolidation therapy with durvalumab after concurrent or sequential chemoradiotherapy. The primary end points were investigator-assessed real-word PFS (rwPFS) and OS. After a median follow-up of 23.5 months, durvalumab achieved a median rwPFS of 21.7 months and 3-year OS rate of 63.2%. In line with the results of Gemstone-301, survival benefit could also be achieved in patients treated with sequential chemoradiotherapy, with a median rwPFS of 19.3 months; rwPFS was longer in patients with PD-L1 expression ≥ 1% versus < 1% (22.4 vs. 15.6 months) [58].

Based on the aforementioned results, we summarize the current and future changes in the treatment of unresectable stage III NSCLC patients, including those based on trials that led to drug approval (Fig. 1). The ongoing trials are summarized in Table 2.

### Unresolved clinical issues

The role of induction therapy in unresectable stage III NSCLC is unclear. Neoadjuvant chemotherapy has moderate efficacy in disease downstaging (reduction of disease stage from baseline), with a pCR rate of only 5–10% [59, 60]. Treatment with ICIs improved the pCR rate to 10–30% and MPR rate to 30–50% [35, 36, 61]. The Checkmate816 trial showed greater pathological regression (pCR rate: 24.0% vs. 2.2%), clinical response (OR: 54% vs. 37%), and radiographic downstaging (31% vs. 24%) in the nivolumab plus chemotherapy group compared with the placebo group [29]. Thus, neoadjuvant therapy may convert disease status from unresectable to resectable in patients with stage III NSCLC. However, most clinical trials did not administer induction therapy for unresectable stage III patients. A phase II study (NCT04580498), enrolling 107 patients, found that SHR-1701, a PD-L1/TGF-β antibody, is safe and effective for unresectable stage III NSCLC [62]. The patients were treated with induction therapy involving three cycles of SHR-1701 with or without chemotherapy before surgery or definitive chemoradiotherapy, followed by maintenance therapy with 16 cycles of SHR-1701. The post-induction and best overall objective response (ORR) rates were 56.1% and 70.1%, respectively. The median EFS was 18.2 months. In accordance with the decision of the multidisciplinary team, one-fourth of the patients received surgery; these patients had MPR and pCR rates of 44.4% and 25.9%, respectively; the median EFS was not reached. These results suggest that some patients may develop resectable disease from unresectable disease and have improved survival. A similar ongoing study, APPRAOCH/CTONG2101, used almonertinib as induction and maintenance therapy for *EGFR*-mutant stage III NSCLC [63]; its results are awaiting.

The optimal management of unresectable stage III NSCLC with actionable genetic alterations is unclear. In the PACIFIC trial, patients were selected based on their genomic profile. Forty-three patients with *EGFR*-mutant NSCLC were included in the durvalumab (*n* = 29) and placebo (*n* = 14) groups. Subgroup analysis according to the *EGFR* mutation status failed to show significant differences due to the small sample size [54]. In a retrospective study by Hellyer et al. [47], stage III NSCLC patients were treated with durvalumab as consolidation therapy following definitive chemoradiation. Patients with *EGFR* or human epidermal growth factor receptor 2 (*HER2*) mutations had a shorter DFS compared with patients with the wild-type genes (7.5 months vs. not reached; *p* = 0.04). These results suggest that locally advanced NSCLC with *EGFR* or *HER2* mutations are unlikely to benefit from ICIs [64] because of the uninflamed tumor microenvironment [65]. Therefore, NSCLC with actionable genes should be treated with durvalumab cautiously. LAURA is a phase III study comparing osimertinib with placebo following chemoradiotherapy for *EGFR*-mutant NSCLC [66]. A phase I–III umbrella study (NCT05170204) evaluated the efficacy and safety of an ALK-TKI, ROS1-TKI, and rearranged during transfection–TKI in patients with unresectable stage III NSCLC according to their biomarker status [67].

It is unclear whether the new treatments should be added during chemoradiotherapy or maintenance therapy for stage III NSCLC. Durvalumab and sugemalimab should be administered as maintenance therapy after definitive chemoradiotherapy. Most trials, such as PACIFIC-8, PACIFIC-9, and SKYSCRAPER-03, have followed the study design of the PACIFIC study using novel agents or combination therapy with durvalumab administered to the control arm [68–70]. Some clinical trials, such as KEYVIBE-006, KEYLYNK-012, and CheckMate73L, added a PD-1 inhibitor or another ICI to concurrent chemoradiotherapy and used monotherapy or combined therapy as maintenance therapy [71–73]. Further studies are needed to determine the toxicity of these agents with chemoradiotherapy.

## Advanced-stage NSCLC

**Fig. 2:**
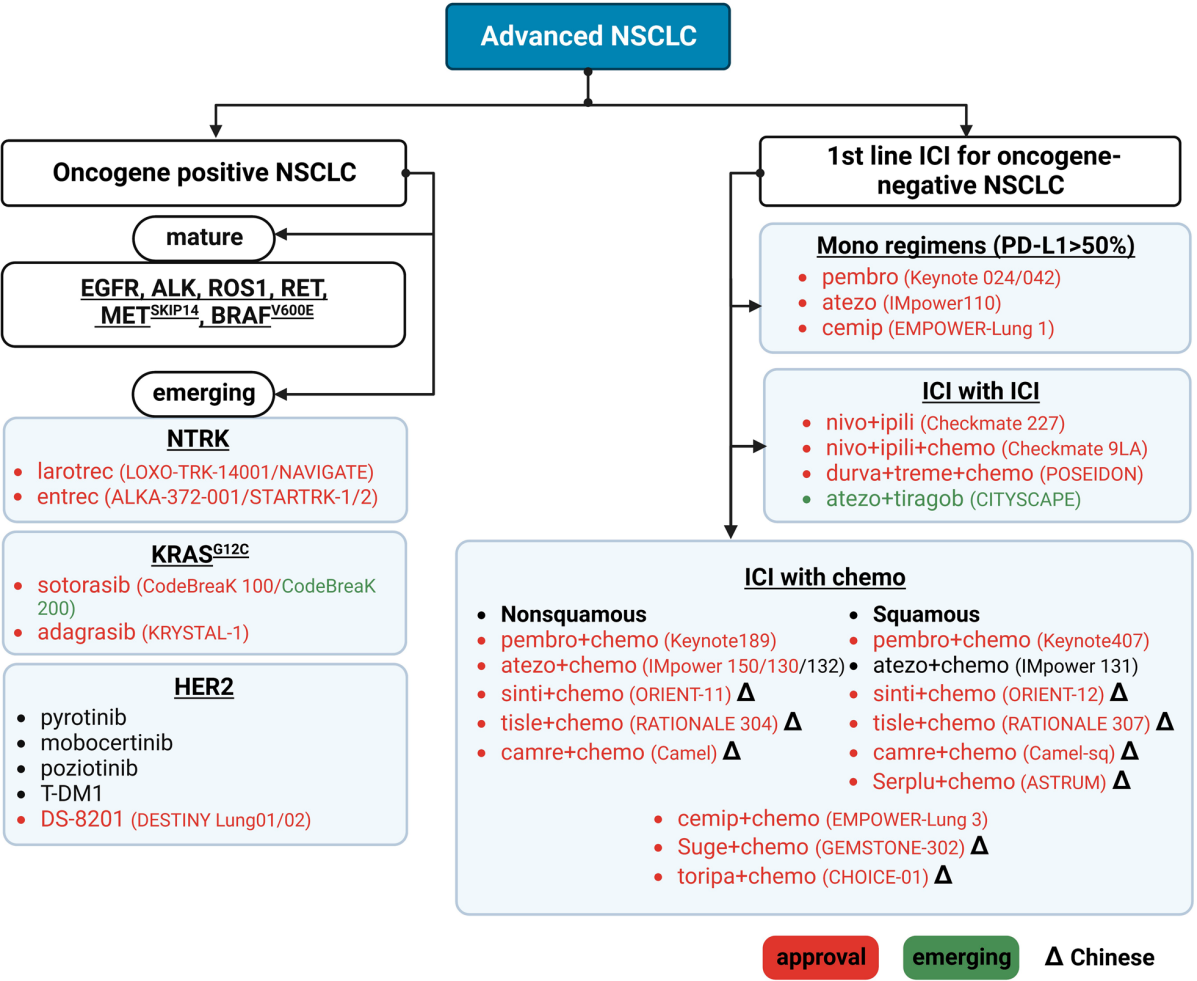
Treatment algorithm for advanced NSCLC. Larotrec, larotrectinib; entrec, entrectinib; pembro, pembrolizumab; atezo, atezolizumab; cemip, cemiplimab; toripa, toripalimab; nivo, nivolumab; ipili, ipilimumab; durva, durvalumab; atezo, atezolizumab; tirago, tiragolumab; treme, tremelimumab; sinti, sintilimab; tisle, tislelizumab; camre, camrelizumab; serplu, serplulimab; suge, sugemalimab; chemo, chemotherapy

Approximately 60% of patients with NSCLC have locally advanced or advanced disease at the time of diagnosis [74] and thus are not eligible for curative treatment. Molecular characterization of advanced NSCLC has resulted in an established treatment paradigm that targets well-characterized oncogenes, including *EGFR*, *ALK, ROS1*, *RET*, mesenchymal–epithelial transition factor exon 14 skipping (*MET*^*14 skipping*^), and V-Raf murine sarcoma viral oncogene homolog B p.V600E (*BRAF*^*V600E*^) alterations [75]. Patients without druggable oncogenes are treated with monotherapy or combination therapy with ICIs that target PD-1/PD-L1 [76, 77]. Recently, the range of actionable alterations has been expanded to include Kirsten rat sarcoma viral oncogene homolog G12C (*KRAS*^*G12C*^), neurotrophic tyrosine receptor kinase (*NTRK*) 1, and *HER2* due to the approval of new therapies. The novel ICIs and their combinations are also being used to treat patients with brain metastasis. We discuss emerging evidence that has modified the current treatment paradigm for advanced NSCLC (Fig. 2). We also discuss the main unresolved clinical issues and ongoing clinical trials that may impact clinical practice in the next 5 years (Table 3).

**Table 3:** Ongoing trials of advanced NSCLC that may change clinical practice in 5 years

Oligometastases				
Therapeutic regimens	Status	NCT No	Sample size	Primary endpoint
Standard medical therapy ± surgery/RT	Phase III	NCT03827577 (OMEGA)	195	OS
Chemotherapy/erlotinib/pembrolizumab ± RT	Phase II/III	NCT03137771 (NRG-LU002)	400	PFS/OS
Systemic anti-cancer therapy ± RT	Phase III	NCT02417662 (SARON)	340	OS
Standard of Care ± RT	Phase II/III	NCT02759783 (CORE)	206	PFS
osimertinib ± LCT	Phase II	NCT03410043 (NORTHSTAR)	143	PFS
Brigatinib + LCT	Phase I	NCT03707938 (BRIGHTSTAR)	35	Incidence of adverse events
ipilimumab + nivolumab ± LCT	Phase III	NCT03391869 (LONESTAR)	360	OS

KRAS					
KRAS^G12C^ inhibitors	Drug type	Status	NCT No	Sample size	Primary endpoint
*KRAS inhibitor monotherapy*					
Sotorasib	KRAS G12C inhibitor	Phase III	NCT04303780 (CodeBreaK200)	345	PFS
Adagrasib	KRAS G12C inhibitor	Phase III	NCT04685135 (KRYSTAL-12)	340	PFS
JDQ443	KRAS G12C inhibitor	Phase Ib/II Phase III	NCT04699188 (KontRASt-01) NCT05132075 (KontRASt-02)	425; 360	DLT, AE, SAE, dose interruptions and reductions, dose intensity, ORR; PFS
D-1553	KRAS G12C inhibitor	Phase I/II	NCT04585035 (D1553-101) NCT05383898	200 203	DLT, AE, Plasma concentration; DLT, AE, ORR
GDC-6036	KRAS G12C inhibitor	Phase Ia/b Phase II/III	NCT04449874 (GO42144) NCT03178552 (BFAST-cohort G)	498 1000 (all cohorts)	AE, DLT; PFS
*Combination with KRAS inhibitors*					
Sotorasib + RMC-4630	KRAS G12C; SHP2 inhibitor	Phase II	NCT05054725	46	ORR
Sotorasib/Adagrasib + TNO155	KRAS G12C; SHP2 inhibitor	Phase I/II	NCT04185883 (CodeBreak101) NCT04330664 (KRYSTAL-2)	1054 (all cohorts) 86	DLT, TEAE, AE, vital signs, ECG, laboratory test values, ORR; AE, Plasma concentration
Adagrasib + pembro	KRAS G12C; immune checkpoint inhibitor	Phase II	NCT04613596 (KRYSTAL 7)	250	ORR
Adagrasib + BI1701963	KRAS G12C; SOS1 inhibitor	Phase I/Ib	NCT04975256 (KRYSTAL 14)	100	TRAE, Plasma concentration, DLT

NTRK					
TRK inhibitors	Drug type	Status	NCT No	Sample size	Primary endpoint
Selitrectinib	Next generation TRK inhibitor	Phase I/II	NCT03215511	81 (adult and pediatric patients)	MTD, recommended dose, ORR
Repotrectinib	Next generation TRK /ROS1 inhibitor	Phase I/II	NCT04094610 NCT03093116 (TRIDENT-1)	75 (adult and pediatric patients); 500 (all arms)	DLT, ORR; DLT, RP2D, ORR

HER2					
HER2 inhibitors	Drug type	Status	NCT No	Sample size	Primary endpoint
T-DXd	ADC	Phase II Phase III	NCT04644237 (DESTINY-Lung02) NCT05048797 (DESTINY-Lung04)	150 264	ORR PFS
T-DM1	ADC	Phase II	NCT02675829	140	ORR
A166	ADC	Phase I/II	NCT03602079	49	MTD
Pyrotinib	TKI	Phase III	NCT04447118 (PYRAMID-1)	150	PFS
Poziotinib	TKI	Phase III	NCT05378763 (PINNACLE)	268	PFS

ADC					
ADC agents	ADC targets	Status	NCT No	Sample size	Primary endpoint
HER3-DXd	HER3	Phase II Phase I	NCT04619004 (HERTHENA-Lung01) NCT03260491	420 264	ORR; DLT, AE, ORR, maximum serum concentration, area under the serum concentration–time curve
ABBV-399	C-MET	Phase II Phase III	NCT03539536 NCT04928846	270 698	ORR, AE; PFS, OS
IMMU-132	TROP-2	Phase II Phase III	NCT03964727 (TROPICS-03) NCT05089734	165 520	ORR; OS
DS-1062	TROP-2	Phase III	NCT04656652 (TROPION-LUNG01)	590	PFS, OS
SAR408701	CEACAM5	Phase III	NCT04154956 (CARMEN-LC03)	554	PFS, OS
SAR408701 + ramucirumab	CEACAM5; Immune checkpoint inhibitor	Phase II	NCT04394624 (CARMEN-LC04)	36	DLT, ORR
SAR408701 + pembro	CEACAM5; Immune checkpoint inhibitor	Phase II	NCT04524689 (CARMEN-LC05)	120	DLT

ICI for oncogene-negative NSCLC					
PD-1/PD-L1 inhibitor	Drug type	Status	NCT No	Sample size	Primary endpoint
Atezo + tirago	Anti-PD-L1; anti-TIGIT	Phase III	NCT04294810 (SKYSCRAPER-01)	635	PFS, OS
Pembro + MK-7684a	Anti-PD-1; anti-TIGIT	Phase III	NCT04738487 (MK-7684A-003)	1246	OS (stratified by PD-L1 expression)
Pembro + Eftilagimod alpha	Anti-PD-1; anti-LAG3	Phase II	NCT03625323 (TACTI-002)	189	ORR
Pembro + Lenvatinib + chemo	Anti-PD-1; anti-VEGF	Phase III	NCT03829319 (LEAP-006)	726	DLT, AE, PFS, OS
Pembro + Lenvatinib	Anti-PD-1; anti-VEGF	Phase III	NCT03976375 (LEAP-008)	405	OS, PFS

*RT* radiotherapy, *LCT* local consolidation therapies, *PFS* progression-free survival, *OS* overall survival, *DLT* dose-limiting toxicity, *AE* adverse event, *SAE* serious adverse event, *ORR* objective response rate, *TEAE* treatment emergent adverse events, *MTD* maximum tolerated dose, *RP2D* recommended phase II dose, *ADC* antibody-drug conjugate

### Oligometastases

#### Emerging evidence with clinical implications

The oligometastasis hypothesis, initially proposed by Hellman and Weichselbaum in 1995 [78], suggests a restricted metastatic state. Oligometastases were traditionally defined based on imaging features and the number and size of metastases: no more than five metastatic sites with an indolent biology [79, 80]. However, this definition lacked specificity, which led to significant heterogeneity in the prognosis and 5-year OS rate (8.3–86%) of NSCLC [81].

Recently, consensus between the European Society for Therapeutic Radiology and Oncology (ESTRO) and European Organisation for Research and Treatment of Cancer (EORTC) [82], as well as consensus between the ESTRO and American Society of Radiation Oncology (ASTRO) [83], suggested that the spectrum of oligometastases should include distinct states based on clinical characterizations, including time to first detection and treatment response. The new classification incorporated independent prognostic factors and is being evaluated in prospective studies (ESTRO, EORTC, and OligoCare) [84]. But the oligometastatic spectrum should still be classified from the perspective of precision medicine based on the underlying biological characteristics of the tumor and immune contexture.

Recent evidence from several phase II randomized controlled trials of NSCLC patients suggests that the addition of local consolidation therapy (LCT) to systemic therapy for oligometastatic NSCLC prolongs the PFS and OS and may even be curative for some patients [80]. Gomez et al. [85, 86] found that local therapy (radiotherapy or surgery) administered after induction chemotherapy prolonged the median PFS (14.2 vs. 4.4 months; *p* = 0.022) and OS (41.2 vs. 17.0 months; *p* = 0.017) compared with those without LCT. The median time to appearance of new metastasis was higher in the LCT group than the control group (11.9 vs. 5.7 months; *p* = 0.0497). Similar to Gomez et al. [85, 86], Iyengar et al. [87] found beneficial effects of LCT after systemic chemotherapy. Another study of *EGFR*-mutant NSCLC patients administered first-generation EGFR-TKIs and upfront radiotherapy for both the primary and metastatic sites reported a significantly prolonged median PFS (20.2 vs. 12.5 months; *p* < 0.001) and OS (25.5 vs. 17.4 months; *p* < 0.001) compared with those without radiotherapy [88]. Based on these results, several guidelines, including those published by European Society of Medical Oncology and The National Comprehensive Cancer Network, have recommended surgery or radiotherapy for the management of oligometastatic disease [89, 90]. New treatment paradigm was thus established in this regard.

#### Unresolved clinical issues

It is unclear whether any specific patient subgroups benefit from LCTs. The benefit of local therapy was observed in oligometastatic cancers of various histological subtypes in the phase II SABR COMET trial [91]. By contrast, another phase II trial of breast cancer (CURB) showed no effect of stereotactic body radiotherapy on PFS in oligoprogressive disease, whereas a substantial response was observed in the NSCLC cohort [92]. Therefore, predictive biomarkers of the response to LCTs are needed. Advancements in liquid biopsy, imaging technology, and tumor biology may allow the role of LCT to be evaluated in different oligometastatic states and determination of the potential safety of de-escalation or omission of systemic therapy. Results from confirmatory randomized trials are expected to prompt changes in future clinical guidelines (Table 3). Furthermore, because TKIs (such as osimertinib and alectinib for *EGFR*/*ALK-*positive patients) and ICIs (for driver mutation-negative patients) have shown durable control of lung cancer, further studies are required to explore the effectiveness of local therapy with newer systemic therapies for oligometastases. Several ongoing trials have explored these unresolved issues, and their results are awaited (Table 3).

### KRAS^G12C^

#### Emerging evidence with clinical implications

*KRAS*, a mutated oncogene, was first identified in 1967 and has long been considered “undruggable” [93]. *KRAS* mutations occur in 25–30% of lung adenocarcinomas. The G12C variant (i.e., change from glycine to cysteine) accounts for 39% of *KRAS* mutations, whereas the G12D variant (i.e., change from glycine to aspartic acid) accounts for 17% [94–96]. Approximately 10% of Chinese patients with NSCLC have *KRAS* mutations, which is a lower proportion than that in the Western population. Of these patients, nearly 30% have the *KRAS*^*G12C*^ mutation [97]. In 2013, a new pocket was identified in which mutant *KRAS* protein binds to GDP near the effector binding switch II area; the identification of this pocket allows *KRAS*^*G12C*^ to be targeted for the treatment of *KRAS*^*G12C*^-mutant NSCLC [98]. Then, a new stage has been set for the treatment of *KRAS*^*G12C*^-mutated NSCLC.

Two trials have targeted *KRAS*^*G12C*^ using sotorasib and adagrasib. In the phase II CodeBreaK100 trial, 124 *KRAS*^*G12C*^ mutated, previously treated patients with NSCLC were treated with sotorasib and updated results showed an ORR of 41%, median PFS of 6.3 months, and median OS of 12.5 months [99]. Sotorasib was more effective than standard therapy with docetaxel, which prompted the FDA to approve it as the first *KRAS* inhibitor. Furthermore, the phase III CodeBreak 200 trial was initially for confirmatory purpose but failed to do so. It has just reported to meet its primary endpoint of PFS comparing sotorasib with docetaxel (PFS, median 5.6 and 4.5 months, respectively (p = 0.002) [100]. However, there was no difference in the median OS between the two treatment arms (10.6 vs. 11.3 months; *p* = 0.53). Another phase II trial, KRYSTAL-1, found similar results in 116 patients with *KRAS*^*G12C*^-mutant NSCLC [101] who were treated with adagrasib as second- or later-line therapy. The ORR was 42.9%, median PFS was 6.5 months, and median OS was 12.6 months. Thus, adagrasib just became the second *KRAS* inhibitor approved by the FDA. Although sotorasib and adagrasib have similar antitumor effects, intracranial response is still a black box for sotorasib. Adagrasib seemed to step further by presenting its effectiveness in controlling intracranial disease [102]. A retrospective subgroup analysis in KRYSTAL-1 showed an intracranial ORR of 33% and median intracranial PFS of 5.4 months for treated central nervous system (CNS) metastases evaluated using the RANO-BM criteria [101]. A similar intracranial ORR of 32% was observed for untreated CNS metastases [103]. These data suggest comparable control of intracranial and systemic disease by adagrasib. These results, new treatment options of targeted therapy for *KRAS*^*G12C*^ already become available for clinical use and a new era is coming (Fig. 2).

#### Unresolved clinical issues

Although several new *KRAS*^*G12C*^ inhibitors have been developed, some clinical issues remain to be resolved. The results of CodeBreak 200 don’t finally serve its initially confirmatory purpose because of the small magnitude of improvement in OS by sotorasib. This may be because of the heterogeneous genetic characteristics of *KRAS* [104]. The rat sarcoma virus pathway and *KRAS* biology are more complex than receptor tyrosine kinase (RTK) drivers, as indicated by their resistance mechanisms to sotorasib and adagrasib, including secondary *KRAS* mutations, bypass activation of the RTK/RAS signaling pathway, co-existing mutations, histological transformation, and immunological adaptation [105]. These results suggest that monotherapy has limited efficacy. The need to obtain a prolonged response and overcome these resistance mechanisms with acceptable safety highlights the need for more potent *KRAS*^*G12C*^ inhibitors and combination strategies. Until now, at least 13 *KRAS*^*G12C*^ inhibitors have been evaluated in trials. Of these, several (e.g., JDQ443 and GDC-6036) are highly selective and have shown promising results. The combination of *KRAS* inhibitors with other targeted inhibitors may prevent or delay the emergence of resistance by targeting more than one oncogenic pathway. Furthermore, because *KRAS* variants and co-mutations are related to the efficacy of ICIs [106, 107], combinations of *KRAS* inhibitors and ICIs are also being investigated **(**Table 3**).** Some of these strategies may become upfront choices for advanced *KRAS*-driven NSCLC in the future.

### NTRK

#### Emerging evidence with clinical implications

*NTRK* fusion genes were first identified to be oncogenic in colorectal tumors in 1982 [108]. Since then, *NTRK1–3* fusion genes have been identified in several tumor types and are present in < 1% of NSCLC cases [109]. *NTRK* alterations are present in 0.59% of Chinese patients with NSCLC [110]. The identification of *NTRK* as an oncogenic driver has prompted studies of targeted therapy against tyrosine receptor kinase (TRK) fusion genes.

Several clinical trials have shown that *NTRK* fusion genes are sensitive to certain TKIs in adult and pediatric tumors, including NSCLC. The first-generation TRK inhibitors larotrectinib and entrectinib received tumor-agnostic approval by the FDA in 2018 and 2019, respectively. The antitumor activity of larotrectinib was investigated in a pooled analysis of several phase I and II trials of solid tumors. Its efficacy in lung cancers with *TRK* fusion genes was comparable with that in other histological types of cancers. The ORR and median PFS were 73% and 35.4 months for lung cancer [111] and 69% and 29.4 months for solid tumors [112], respectively. Entrectinib also demonstrated a durable systemic response, with an ORR of 61.3% and a median PFS of 13.8 months [113]. Both drugs demonstrated CNS activity. An integrated analysis of patients with lung cancer and CNS metastases showed an ORR of 63% with larotrectinib versus 67% with entrectinib [114–116]. Thus, TRK inhibitors that target *NRTK* fusion genes are effective for the treatment of lung cancers (Fig. 2).

#### Unresolved clinical issues

The treatment paradigm for *NTRK* fusion gene-positive lung cancer is similar to that for cancers expressing other oncogenes. Resistance to TRK inhibitors is inevitable and is mediated by on-target resistance, *NTRK* kinase domain mutations, and off-target resistance, such as *MET* amplification and *BRAF* or *KRAS* mutations [117]. To overcome on-target resistance, next-generation TRK inhibitors are needed. Several new agents are under investigation, of which selitrectinib and repotrectinib had the most promising results in early reports (ORR = 45% vs. 50%) [118, 119] (Table 3). Another agent, PBI-200, has higher brain penetrance; however, the results of its efficacy are awaiting. ICP-723 is a potent next-generation TRK inhibitor developed by Chinese investigators and is highly active against resistance mutations (e.g., *NTRK G595R, F589L,* and *G667C/A/S*) (NCT04685226). Future studies should evaluate strategies to inhibit off-target resistance pathways.

### HER2

#### Emerging evidence with clinical implications

Mutations in the *HER2* oncogene were first identified in 2004 [120] and account for approximately 1–4% of NSCLC cases [121]. The *HER2* alterations that drive tumor development are *HER2* mutations (2–4%), amplification (FISH copy number ≥ 2; 10–20%), and protein overexpression (immunohistochemical score of 2+/3+; 2.4–38%) [122, 123]. The predominant *HER2* mutation in NSCLC is A775_G776insYVMA in exon 20 (80–90%) [124]. Strategies that target *HER2* alterations are effective for other cancers, such as breast cancer; however, they have produced conflicting results in NSCLC.

In the past two decades, several anti-*HER2* therapies, including monoclonal antibodies, chemotherapy, TKIs, and antibody–drug conjugates (ADCs), have been reported. Pan-HER2-TKIs, such as afatinib, neratinib, and dacomitinib, showed no benefit for NSCLC [125–127]. Selective HER2-TKIs, such as pyrotinib, mobocertinib, and poziotinib, had moderate efficacy as second- or later-line therapies, with ORRs of 20–30% [128, 129]. However, inhibition of *HER2* activity by HER2-TKIs is limited by their high toxicity. Thus, HER2-TKIs are used only as second- or later-line treatments. Combination of the *HER2* antibodies trastuzumab and pertuzumab with versus without docetaxel produced a similar response rate of 21–29% [130].

Recently, promising data have been reported for anti-*HER2* ADCs. The first ADC, trastuzumab emtansine (T-DM1), was more effective for lung cancer than were previous treatments that targeted *HER2* mutations, with an ORR of 44% and median PFS of 5 months [131]. Based on the engineering mechanism of T-DM1, trastuzumab deruxtecan (DS-8201) was developed by switching the payload and enabling the bystander effect, leading to the highest clinical activity yet [132]. The phase II DESTINY-Lung01 trial included 91 patients with *HER2*-mutated, treated NSCLC who received DS-8201 and reported an ORR of 55%, median PFS of 8.2 months, and median OS of 17.8 months [133]. Similar responses were observed for all *HER2* mutations regardless of the gene amplification or protein expression status or presence of CNS metastasis. Another phase I trial of DS-8201 demonstrated a higher ORR of 72.7% and a median PFS of 11.3 months in the NSCLC group [134]. Based on the phase II randomized DESTINY-LUNG02 trial, which showed an ORR of 53.8% in previously treated *HER2*-mutant NSCLC (NCT04644237) [135], the FDA has recently granted accelerated approval to DS-8201 for treatment of previously treated *HER2*-mutant NSCLC. Therefore, a new standard of care was established for patients with NSCLC harboring *HER2* mutations (Fig. 2).

#### Unresolved clinical issues

Several clinical issues regarding novel therapies for *HER2*-mutant NSCLC remain unresolved. First, it is unclear whether DS-8201 is effective as first-line treatment. First-line treatment with DS-8201 is being investigated in the DESTINY Lung04 trial (NCT05048797). Second, there is limited evidence on the mechanisms underlying resistance to targeted therapy and ADCs. Therefore, future studies should explore strategies to overcome resistance. Third, the CNS activity of emerging anti-*HER2* drugs was not evaluated in previous trials. However, ongoing trials have included patients with CNS metastases (Table 3).

### ICIs

#### Emerging evidence with clinical implications

ICIs are the standard treatment for patients with oncogene-negative advanced NSCLC, in which PD-L1 expression is the most robust biomarker. Monotherapy with PD-1/PD-L1 inhibitors is approved for patients with ≥ 50% PD-L1 expression. Additionally, combination therapy of PD-1/PD-L1 inhibitors and chemotherapy (bevacizumab or anti-CTLA-4 agents) has been approved as first-line therapy for patients with < 50% PD-L1 expression [76]. However, the response rate was higher and OS longer in patients treated with ICI plus chemotherapy compared with ICI monotherapy among patients with high PD-L1 expression [136]. The following section describes the recent clinical studies.

First, the novel PD-1/PD-L1 drugs provide additional treatment options compared with conventional ICIs [137]. In the EMPOWER-Lung 1 trial, which included patients with a PD-L1 tumor proportion score (TPS) ≥ 50%, first-line therapy with cemiplimab significantly prolonged the median PFS (8.2 vs. 5.0 months) and OS (21.9 vs. 13.0 months) [138]. The phase III EMPOWER-Lung 3 study enrolled advanced NSCLC patients irrespective of their PD-L1 expression level and found that cemiplimab plus chemotherapy was associated with clinically and statistically significant improvements in PFS and OS compared with chemotherapy alone [139]. The combination of toripalimab (CHOICE-01) and sugemalimab (GEMSTONE 302), developed in China, with chemotherapy prolonged the median PFS and OS in treatment-naive, advanced NSCLC patients [140, 141]. Second, new treatment combinations have been investigated to enhance the efficacy of the approved options. The combination of anti-PD-L1 and anti-TIGIT showed promising results. In the phase II CITYSCAPE trial, first-line treatment with tiragolumab (an anti-TIGIT antibody) plus atezolizumab improved the ORR and PFS in patients with metastatic NSCLC and a PD-L1 TPS ≥ 50% [142]. The phase III POSEIDON trial showed that first-line treatment with tremelimumab plus durvalumab and chemotherapy significantly prolonged PFS (6.2 vs. 4.8 months; *p* = 0.0003) and OS (14 vs. 11.7 months; *p* = 0.003) compared with chemotherapy alone [143]. The clinical benefit was highest in patients with non-squamous cell carcinoma and a PD-L1 TPS ≥ 1%. Third, patients with untreated or active brain metastases were often under-represented in previous clinical trials. In the single-arm phase II ATEZO-BRAIN trial, first-line treatment with atezolizumab plus chemotherapy exhibited similar systemic and intracranial response rates (47.5% vs. 40%) as assessed by the RECIST 1.1 and RANO-BM criteria, respectively. The median intracranial PFS was 6.9 months and the median OS 13.6 months, which were not affected by corticosteroid use [144]. Furthermore, the phase III ORIENT-31 trial, which administered a combination of anti-angiogenic therapy and chemotherapy, showed that ICIs are effective for oncogene-positive NSCLC patients who failed prior targeted therapy [145]. Sintilimab plus a bevacizumab biosimilar (IBI305) and chemotherapy prolonged the median PFS (6.9 vs. 4.3 months; *p* < 0.0001) and was well-tolerated in patients with *EGFR*-mutant NSCLC who progressed on previous EGFR-TKI therapy. A subgroup analysis in the IMpower150 trial revealed prolonged OS with a combination of atezolizumab, bevacizumab, carboplatin, and paclitaxel compared with a combination of bevacizumab, carboplatin, and paclitaxel in patients harboring *EGFR* mutations, including those with previous TKI failure. However, those results should be interpreted with caution [146]. Importantly, the first randomized phase III study, CheckMate722, showed negative results, with no significant improvement in PFS after nivolumab plus chemotherapy compared with chemotherapy alone in patients harboring *EGFR* mutations [147]. The aforementioned conflicting data suggest that an anti-angiogenic strategy has an important effect. Recent data have modified the complex treatment paradigm for NSCLC, which includes multiple treatment options, and PD-L1 expression remains an important biomarker (Fig. 2). The long-term outcomes of the new treatment options have confirmed their efficacy and safety. Thus, treatment selection should be careful and sometimes rely on clinical factors and patient preference [148].

#### Unresolved clinical issues

Despite the impressive efficacy of ICIs for NSCLC treatment, failure to respond due to primary or secondary resistance is common. The resistance mechanisms in human cancers include loss of neoantigens, defects in antigen presentation and interferon signaling, upregulation of immune inhibitory molecules, and exclusion of T cells [149–151]. Further translational and biological studies are needed to enhance the efficacy of ICI regimens and overcome resistance (Table 3).

## Conclusions

Molecular-driven treatments that were previously only available for advanced-stage NSCLC have recently been shown to be effective for early- and locally advanced-stage disease. Recent studies have evaluated therapies that target a broader range of oncogenes to overcome drug resistance and treat patients who were previously excluded from clinical trials of advanced NSCLC. Emerging data are likely to impact future treatment guidelines and promote the use of personalized medicine by these ongoing trials. We envision that the treatment landscape will definitely evolve continuously, leading to improved survival and quality of life of patients with lung cancer.

## Data Availability

All data generated or analyzed during this study are included in this published article.

## Abbreviations

ADCs: Anti-body-drug conjugates
ALK: Anaplastic lymphoma kinase
ASTRO: American Society of Radiation Oncology
BRAFV600E: V-Raf murine sarcoma viral oncogene homolog B p.V600E
CNS: Central nervous system
ctDNA: Circulating tumor DNA
DFS: Disease-free survival
DS-8201: Trastuzumab deruxtecan
EFS: Even-free survival
EGFR: Epidermal growth factor receptor
ESTRO-EORTC: European Society for Therapeutic Radiology and Oncology–European Organisation for Research and Treatment of Cancer
FDA: Food and Drug Administration
HER2: Human epidermal growth factor receptor 2
HR: Hazard ratio
ICIs: Immune checkpoint inhibitors
KRASG12C: Kirsten rat sarcoma viral oncogene homolog G12C
LCT: Local consolidation therapies
MET14 skipping: Mesenchymal-epithelial transition factor exon 14 skipping
MRD: Molecular residual disease
NSCLC: Non-small cell lung cancer
NTRK1: Neurotrophic tyrosine receptor kinase
OS: Overall survival
pCR: Pathological complete response
PD-1/PD-L1: Programmed death 1/programmed death ligand 1
PFS: Progression-free survival
RAS: Rat sarcoma virus
RET: Rearranged during transfection
ROS1: C-ros oncogene 1
RTK: Receptor tyrosine kinase
TC: Tumor cells
T-DM1: Trastuzumab emtansine
TKIs: Tyrosine kinase inhibitor
TRK: Tyrosine receptor kinase
WCLC: World Conference of Lung Cancer

## References

[1] Siegel RL, Miller KD, Fuchs HE, Jemal A (2022). Cancer statistics, 2022. CA Cancer J Clin.

[2] Pao W, Chmielecki J (2010). Rational, biologically based treatment of EGFR-mutant non-small-cell lung cancer. Nat Rev Cancer.

[3] Mok TS, Wu YL, Thongprasert S, Yang CH, Chu DT, Saijo N, Sunpaweravong P, Han B, Margono B, Ichinose Y, Nishiwaki Y, Ohe Y, Yang JJ, Chewaskulyong B, Jiang H, Duffield EL, Watkins CL, Armour AA, Fukuoka M (2009). Gefitinib or carboplatin-paclitaxel in pulmonary adenocarcinoma. N Engl J Med.

[4] Zhou C, Wu YL, Chen G, Feng J, Liu XQ, Wang C, Zhang S, Wang J, Zhou S, Ren S, Lu S, Zhang L, Hu C, Hu C, Luo Y, Chen L, Ye M, Huang J, Zhi X, Zhang Y, Xiu Q, Ma J, Zhang L, You C (2011). Erlotinib versus chemotherapy as first-line treatment for patients with advanced EGFR mutation-positive non-small-cell lung cancer (OPTIMAL, CTONG-0802): a multicentre, open-label, randomised, phase 3 study. Lancet Oncol.

[5] Wu YL, Planchard D, Lu S, Sun H, Yamamoto N, Kim DW, Tan D, Yang JC, Azrif M, Mitsudomi T, Park K, Soo RA, Chang J, Alip A, Peters S, Douillard JY (2019). Pan-Asian adapted Clinical Practice Guidelines for the management of patients with metastatic non-small-cell lung cancer: a CSCO-ESMO initiative endorsed by JSMO, KSMO, MOS, SSO and TOS. Ann Oncol.

[6] Ramalingam SS, Vansteenkiste J, Planchard D, Cho BC, Gray JE, Ohe Y, Zhou C, Reungwetwattana T, Cheng Y, Chewaskulyong B, Shah R, Cobo M, Lee KH, Cheema P, Tiseo M, John T, Lin MC, Imamura F, Kurata T, Todd A, Hodge R, Saggese M, Rukazenkov Y, Soria JC (2020). Overall survival with osimertinib in untreated, EGFR-mutated advanced NSCLC. N Engl J Med.

[7] Chen R, Manochakian R, James L, Azzouqa AG, Shi H, Zhang Y, Zhao Y, Zhou K, Lou Y (2020). Emerging therapeutic agents for advanced non-small cell lung cancer. J Hematol Oncol.

[8] Boussiotis VA (2016). Molecular and biochemical aspects of the PD-1 checkpoint pathway. N Engl J Med.

[9] Liu S, Wu Y (2018). An immunological storm for cancer therapy: 2018 Nobel Prize in Physiology or Medicine. Sci Bull.

[10] Gettinger S, Horn L, Jackman D, Spigel D, Antonia S, Hellmann M, Powderly J, Heist R, Sequist LV, Smith DC, Leming P, Geese WJ, Yoon D, Li A, Brahmer J (2018). Five-year follow-up of nivolumab in previously treated advanced non-small-cell lung cancer: results from the CA209-003 study. J Clin Oncol.

[11] Reck M, Rodríguez-Abreu D, Robinson AG, Hui R, Csőszi T, Fülöp A, Gottfried M, Peled N, Tafreshi A, Cuffe S, O'Brien M, Rao S, Hotta K, Leal TA, Riess JW, Jensen E, Zhao B, Pietanza MC, Brahmer JR (2021). Five-year outcomes with pembrolizumab versus chemotherapy for metastatic non-small-cell lung cancer with PD-L1 tumor proportion score ≥ 50. J Clin Oncol.

[12] Spigel DR, Faivre-Finn C, Gray JE, Vicente D, Planchard D, Paz-Ares L, Vansteenkiste JF, Garassino MC, Hui R, Quantin X, Rimner A, Wu YL, Özgüroğlu M, Lee KH, Kato T, de Wit M, Kurata T, Reck M, Cho BC, Senan S, Naidoo J, Mann H, Newton M, Thiyagarajah P, Antonia SJ (2022). Five-year survival outcomes from the PACIFIC Trial: durvalumab after chemoradiotherapy in stage III non-small-cell lung cancer. J Clin Oncol.

[13] Zhang C, Leighl NB, Wu YL, Zhong WZ (2019). Emerging therapies for non-small cell lung cancer. J Hematol Oncol.

[14] Pignon JP, Tribodet H, Scagliotti GV, Douillard JY, Shepherd FA, Stephens RJ, Dunant A, Torri V, Rosell R, Seymour L, Spiro SG, Rolland E, Fossati R, Aubert D, Ding K, Waller D, Le Chevalier T (2008). Lung adjuvant cisplatin evaluation: a pooled analysis by the LACE Collaborative Group. J Clin Oncol.

[15] Liu SY, Liu SM, Zhong WZ, Wu YL (2022). Targeted therapy in early stage non-small cell lung cancer. Curr Treat Options Oncol.

[16] Wu YL, Tsuboi M, He J, John T, Grohe C, Majem M, Goldman JW, Laktionov K, Kim SW, Kato T, Vu HV, Lu S, Lee KY, Akewanlop C, Yu CJ, de Marinis F, Bonanno L, Domine M, Shepherd FA, Zeng L, Hodge R, Atasoy A, Rukazenkov Y, Herbst RS (2020). Osimertinib in resected EGFR-mutated non-small-cell lung cancer. N Engl J Med.

[17] Tsuboi M, Wu Y, Grohe C, John T, Tarruella MM, Wang J, Kato T, Goldman JW, Kim S, Yu C, Vu HV, Mukhametshina G, Akewanlop C, de Marinis F, Shepherd FA, Urban D, Stachowiak M, Bolanos AL, Huang X, Herbst RS (2022). LBA47 Osimertinib as adjuvant therapy in patients (pts) with resected EGFR-mutated (EGFRm) stage IB-IIIA non-small cell lung cancer (NSCLC): updated results from ADAURA. Ann Oncol.

[18] Wu YL, John T, Grohe C, Majem M, Goldman JW, Kim SW, Kato T, Laktionov K, Vu HV, Wang Z, Lu S, Lee KY, Akewanlop C, Yu CJ, de Marinis F, Bonanno L, Domine M, Shepherd FA, Zeng L, Atasoy A, Herbst RS, Tsuboi M (2022). Postoperative chemotherapy use and outcomes from ADAURA: osimertinib as adjuvant therapy for resected EGFR-mutated NSCLC. J Thorac Oncol.

[19] FDA approves osimertinib as adjuvant therapy for non-small cell lung cancer with EGFR mutations. https://www.fda.gov/drugs/resources-information-approved-drugs/fda-approves-osimertinib-adjuvant-therapy-non-small-cell-lung-cancer-egfr-mutations 2020.

[20] Zhong WZ, Wang Q, Mao WM, Xu ST, Wu L, Shen Y, Liu YY, Chen C, Cheng Y, Xu L, Wang J, Fei K, Li XF, Li J, Huang C, Liu ZD, Xu S, Chen KN, Xu SD, Liu LX, Yu P, Wang BH, Ma HT, Yan HH, Yang XN, Zhou Q, Wu YL (2018). Gefitinib versus vinorelbine plus cisplatin as adjuvant treatment for stage II–IIIA (N1–N2) EGFR-mutant NSCLC (ADJUVANT/CTONG1104): a randomised, open-label, phase 3 study. Lancet Oncol.

[21] Zhong WZ, Wang Q, Mao WM, Xu ST, Wu L, Wei YC, Liu YY, Chen C, Cheng Y, Yin R, Yang F, Ren SX, Li XF, Li J, Huang C, Liu ZD, Xu S, Chen KN, Xu SD, Liu LX, Yu P, Wang BH, Ma HT, Yang JJ, Yan HH, Yang XN, Liu SY, Zhou Q, Wu YL (2021). Gefitinib versus vinorelbine plus cisplatin as adjuvant treatment for stage II–IIIA (N1–N2) EGFR-mutant NSCLC: final overall survival analysis of CTONG1104 phase III trial. J Clin Oncol.

[22] He J, Su C, Liang W, Xu S, Wu L, Fu X, Zhang X, Ge D, Chen Q, Mao W, Xu L, Chen C, Hu B, Shao G, Hu J, Zhao J, Liu X, Liu Z, Wang Z, Xiao Z, Gong T, Lin W, Li X, Ye F, Liu Y, Ma H, Huang Y, Zhou J, Wang Z, Fu J, Ding L, Mao L, Zhou C (2021). Icotinib versus chemotherapy as adjuvant treatment for stage II–IIIA EGFR-mutant non-small-cell lung cancer (EVIDENCE): a randomised, open-label, phase 3 trial. Lancet Respir Med.

[23] Tada H, Mitsudomi T, Misumi T, Sugio K, Tsuboi M, Okamoto I, Iwamoto Y, Sakakura N, Sugawara S, Atagi S, Takahashi T, Hayashi H, Okada M, Inokawa H, Yoshioka H, Takahashi K, Higashiyama M, Yoshino I, Nakagawa K (2022). Randomized phase III study of gefitinib versus cisplatin plus vinorelbine for patients with resected stage II–IIIA non-small-cell lung cancer with EGFR mutation (IMPACT). J Clin Oncol.

[24] Felip E, Altorki N, Zhou C, Csőszi T, Vynnychenko I, Goloborodko O, Luft A, Akopov A, Martinez-Marti A, Kenmotsu H, Chen YM, Chella A, Sugawara S, Voong D, Wu F, Yi J, Deng Y, McCleland M, Bennett E, Gitlitz B, Wakelee H (2021). Adjuvant atezolizumab after adjuvant chemotherapy in resected stage IB-IIIA non-small-cell lung cancer (IMpower010): a randomised, multicentre, open-label, phase 3 trial. Lancet.

[25] FDA approves atezolizumab as adjuvant treatment for non-small cell lung cancer. https://www.fda.gov/drugs/resources-information-approved-drugs/fda-approves-atezolizumab-adjuvant-treatment-non-small-cell-lung-cancer 2021.

[26] Wakelee H, Altorki N, Felip E, Vallieres E, Vynnychenko IO, Akopov A, Martinez-Marti A, Chella A, Bondarenko I, Sugawara S, Fan Y, Kenmotsu H, Chen YM, Deng Y, Wu F, McNally V, Bennett E, Gitlitz BJ, Zhou C (2022). PL03.09 IMpower010: overall survival interim analysis of a phase III study of atezolizumab vs best supportive care in resected NSCLC. J Thorac Oncol.

[27] O'Brien M, Paz-Ares L, Marreaud S, Dafni U, Oselin K, Havel L, Esteban E, Isla D, Martinez-Marti A, Faehling M, Tsuboi M, Lee JS, Nakagawa K, Yang J, Samkari A, Keller SM, Mauer M, Jha N, Stahel R, Besse B, Peters S (2022). Pembrolizumab versus placebo as adjuvant therapy for completely resected stage IB-IIIA non-small-cell lung cancer (PEARLS/KEYNOTE-091): an interim analysis of a randomised, triple-blind, phase 3 trial. Lancet Oncol.

[28] FDA approves pembrolizumab as adjuvant treatment for non-small cell lung cancer. https://www.fda.gov/drugs/resources-information-approved-drugs/fda-approves-pembrolizumab-adjuvant-treatment-non-small-cell-lung-cancer 2023.

[29] Forde PM, Spicer J, Lu S, Provencio M, Mitsudomi T, Awad MM, Felip E, Broderick SR, Brahmer JR, Swanson SJ, Kerr K, Wang C, Ciuleanu TE, Saylors GB, Tanaka F, Ito H, Chen KN, Liberman M, Vokes EE, Taube JM, Dorange C, Cai J, Fiore J, Jarkowski A, Balli D, Sausen M, Pandya D, Calvet CY, Girard N (2022). Neoadjuvant nivolumab plus chemotherapy in resectable lung cancer. N Engl J Med.

[30] FDA approves neoadjuvant nivolumab and platinum-doublet chemotherapy for early-stage non-small cell lung cancer. https://www.fda.gov/drugs/resources-information-approved-drugs/fda-approves-neoadjuvant-nivolumab-and-platinum-doublet-chemotherapy-early-stage-non-small-cell-lung 2022.

[31] Liu SM, Jie G, Wu Y (2022). Toward a cure for lung cancer: important advances in operable non-small cell lung cancer. Sci Bull.

[32] Efficacy and Safety of Almonertinib Combined With or Without Chemotherapy as an Adjuvant Treatment for Stage II–IIIA Non-small Cell Lung Carcinoma Following Complete Tumour Resection (APEX). https://clinicaltrials.gov/ct2/show/NCT04762459 2021.

[33] Saw S, Ong BH, Chua K, Takano A, Tan D (2021). Revisiting neoadjuvant therapy in non-small-cell lung cancer. Lancet Oncol.

[34] Zhou Z, Ding Z, Yuan J, Shen S, Jian H, Tan Q, Yang Y, Chen Z, Luo Q, Cheng X, Yu Y, Niu X, Qian L, Chen X, Gu L, Liu R, Ma S, Huang J, Chen T, Li Z, Ji W, Song L, Shen L, Jiang L, Yu Z, Zhang C, Tai Z, Wang C, Chen R, Carbone DP, Xia X, Lu S (2022). Homologous recombination deficiency (HRD) can predict the therapeutic outcomes of immuno-neoadjuvant therapy in NSCLC patients. J Hematol Oncol.

[35] Shu CA, Gainor JF, Awad MM, Chiuzan C, Grigg CM, Pabani A, Garofano RF, Stoopler MB, Cheng SK, White A, Lanuti M, D'Ovidio F, Bacchetta M, Sonett JR, Saqi A, Rizvi NA (2020). Neoadjuvant atezolizumab and chemotherapy in patients with resectable non-small-cell lung cancer: an open-label, multicentre, single-arm, phase 2 trial. Lancet Oncol.

[36] Provencio M, Nadal E, Insa A, García-Campelo MR, Casal-Rubio J, Dómine M, Majem M, Rodríguez-Abreu D, Martínez-Martí A, De Castro CJ, Cobo M, López VG, Del BE, Bernabé CR, Viñolas N, Barneto AI, Viteri S, Pereira E, Royuela A, Casarrubios M, Salas AC, Parra ER, Wistuba I, Calvo V, Laza-Briviesca R, Romero A, Massuti B, Cruz-Bermúdez A (2020). Neoadjuvant chemotherapy and nivolumab in resectable non-small-cell lung cancer (NADIM): an open-label, multicentre, single-arm, phase 2 trial. Lancet Oncol.

[37] Girard N, Spicer J, Provencio M, Lu S, Broderick S, Awad MM, Mitsudomi T, Kerr K, Brahmer J, Swanson SJ, Felip E, Wang C, Saylors GB, Chen K, Tanaka F, Liberman M, Dorange C, Mahmood J, Cai J, Forde PM (2022). Abstract CT012: Nivolumab (NIVO) + platinum-doublet chemotherapy (chemo) vs chemo as neoadjuvant treatment for resectable (IB-IIIA) non-small cell lung cancer (NSCLC): event-free survival (EFS) results from the phase 3 CheckMate 816 trial. Cancer Res.

[38] Pantel K, Alix-Panabières C (2019). Liquid biopsy and minimal residual disease - latest advances and implications for cure. Nat Rev Clin Oncol.

[39] Chen K, Zhao H, Shi Y, Yang F, Wang LT, Kang G, Nie Y, Wang J (2019). Perioperative dynamic changes in circulating tumor DNA in patients with lung cancer (DYNAMIC). Clin Cancer Res.

[40] Zhang JT, Liu SY, Gao W, Liu SM, Yan HH, Ji L, Chen Y, Gong Y, Lu HL, Lin JT, Yin K, Jiang BY, Nie Q, Liao RQ, Dong S, Guan Y, Dai P, Zhang XC, Yang JJ, Tu HY, Xia X, Yi X, Zhou Q, Zhong WZ, Yang XN, Wu YL (2022). Longitudinal undetectable molecular residual disease defines potentially cured population in localized non-small cell lung cancer. Cancer Discov.

[41] Chaudhuri AA, Chabon JJ, Lovejoy AF, Newman AM, Stehr H, Azad TD, Khodadoust MS, Esfahani MS, Liu CL, Zhou L, Scherer F, Kurtz DM, Say C, Carter JN, Merriott DJ, Dudley JC, Binkley MS, Modlin L, Padda SK, Gensheimer MF, West RB, Shrager JB, Neal JW, Wakelee HA, Loo BJ, Alizadeh AA, Diehn M (2017). Early detection of molecular residual disease in localized lung cancer by circulating tumor DNA Profiling. Cancer Discov.

[42] Moding EJ, Liu Y, Nabet BY, Chabon JJ, Chaudhuri AA, Hui AB, Bonilla RF, Ko RB, Yoo CH, Gojenola L, Jones CD, He J, Qiao Y, Xu T, Heymach JV, Tsao A, Liao Z, Gomez DR, Das M, Padda SK, Ramchandran KJ, Neal JW, Wakelee HA, Loo BJ, Lin SH, Alizadeh AA, Diehn M (2020). Circulating tumor DNA dynamics predict benefit from consolidation immunotherapy in locally advanced non-small cell lung cancer. Nat Cancer.

[43] Dong S, Wang Z, Zhou Q, Yang L, Zhang J, Chen Y, Liu S, Lin J, Liao R, Tu H, Xu C, Yang X, Zhong W, Yang J, Wu Y (2021). P49.01 Drug holiday based on minimal residual disease status after local therapy following EGFR-TKI treatment for patients with advanced NSCLC. J Thorac Oncol.

[44] Adjuvant Treatment Based on MRD for EGFR Mutant NSCLC. https://clinicaltrials.gov/ct2/show/NCT05536505 2022.

[45] Gou LY, Wu YL (2014). Prevalence of driver mutations in non-small-cell lung cancers in the People's Republic of China. Lung Cancer (Auckl).

[46] Gainor JF, Shaw AT, Sequist LV, Fu X, Azzoli CG, Piotrowska Z, Huynh TG, Zhao L, Fulton L, Schultz KR, Howe E, Farago AF, Sullivan RJ, Stone JR, Digumarthy S, Moran T, Hata AN, Yagi Y, Yeap BY, Engelman JA, Mino-Kenudson M (2016). EGFR mutations and ALK rearrangements are associated with low response rates to PD-1 pathway blockade in non-small cell lung cancer: a retrospective analysis. Clin Cancer Res.

[47] Hellyer JA, Aredo JV, Das M, Ramchandran K, Padda SK, Neal JW, Wakelee HA (2021). Role of consolidation durvalumab in patients with EGFR- and HER2-mutant unresectable stage III NSCLC. J Thorac Oncol.

[48] Zhou C, Kim SW, Reungwetwattana T, Zhou J, Zhang Y, He J, Yang JJ, Cheng Y, Lee SH, Bu L, Xu T, Yang L, Wang C, Liu T, Morcos PN, Lu Y, Zhang L (2019). Alectinib versus crizotinib in untreated Asian patients with anaplastic lymphoma kinase-positive non-small-cell lung cancer (ALESIA): a randomised phase 3 study. Lancet Respir Med.

[49] Shaw AT, Riely GJ, Bang YJ, Kim DW, Camidge DR, Solomon BJ, Varella-Garcia M, Iafrate AJ, Shapiro GI, Usari T, Wang SC, Wilner KD, Clark JW, Ou SI (2019). Crizotinib in ROS1-rearranged advanced non-small-cell lung cancer (NSCLC): updated results, including overall survival, from PROFILE 1001. Ann Oncol.

[50] Drilon A, Oxnard GR, Tan D, Loong H, Johnson M, Gainor J, McCoach CE, Gautschi O, Besse B, Cho BC, Peled N, Weiss J, Kim YJ, Ohe Y, Nishio M, Park K, Patel J, Seto T, Sakamoto T, Rosen E, Shah MH, Barlesi F, Cassier PA, Bazhenova L, De Braud F, Garralda E, Velcheti V, Satouchi M, Ohashi K, Pennell NA, Reckamp KL, Dy GK, Wolf J, Solomon B, Falchook G, Ebata K, Nguyen M, Nair B, Zhu EY, Yang L, Huang X, Olek E, Rothenberg SM, Goto K, Subbiah V (2020). Efficacy of selpercatinib in RET fusion-positive non-small-cell lung cancer. N Engl J Med.

[51] A Study Comparing Adjuvant Alectinib Versus Adjuvant Platinum-Based Chemotherapy in Patients With ALK Positive Non-Small Cell Lung Cancer. https://clinicaltrials.gov/ct2/show/NCT03456076 2018.

[52] Chansky K, Detterbeck FC, Nicholson AG, Rusch VW, Vallières E, Groome P, Kennedy C, Krasnik M, Peake M, Shemanski L, Bolejack V, Crowley JJ, Asamura H, Rami-Porta R (2017). The IASLC lung cancer staging project: external validation of the revision of the TNM stage groupings in the eighth edition of the TNM classification of lung cancer. J Thorac Oncol.

[53] Evison M (2020). The current treatment landscape in the UK for stage III NSCLC. Br J Cancer.

[54] Antonia SJ, Villegas A, Daniel D, Vicente D, Murakami S, Hui R, Yokoi T, Chiappori A, Lee KH, de Wit M, Cho BC, Bourhaba M, Quantin X, Tokito T, Mekhail T, Planchard D, Kim YC, Karapetis CS, Hiret S, Ostoros G, Kubota K, Gray JE, Paz-Ares L, de Castro CJ, Wadsworth C, Melillo G, Jiang H, Huang Y, Dennis PA, Özgüroğlu M (2017). Durvalumab after chemoradiotherapy in stage III non-small-cell lung cancer. N Engl J Med.

[55] FDA approves durvalumab after chemoradiation for unresectable stage III NSCLC. https://www.fda.gov/drugs/resources-information-approved-drugs/fda-approves-durvalumab-after-chemoradiation-unresectable-stage-iii-nsclc 2018.

[56] Zhou Q, Chen M, Jiang O, Pan Y, Hu D, Lin Q, Wu G, Cui J, Chang J, Cheng Y, Huang C, Liu A, Yang N, Gong Y, Zhu C, Ma Z, Fang J, Chen G, Zhao J, Shi A, Lin Y, Li G, Liu Y, Wang D, Wu R, Xu X, Shi J, Liu Z, Cui N, Wang J, Wang Q, Zhang R, Yang J, Wu YL (2022). Sugemalimab versus placebo after concurrent or sequential chemoradiotherapy in patients with locally advanced, unresectable, stage III non-small-cell lung cancer in China (GEMSTONE-301): interim results of a randomised, double-blind, multicentre, phase 3 trial. Lancet Oncol.

[57] Wu YL, Zhou Q, Chen M, Pan Y, Jian O, Hu D, Lin Q, Wu G, Cui J, Chang J, Cheng Y, Huang C, Liu A, Yang N, Gong Y, Zhu C, Ma Z, Fang J, Chen G, Zhao J, Shi A, Lin Y, Li G, Liu Y, Wang D, Wu R, Xu X, Shi J, Liu Z, Wang J, Yang J (2022). OA02.05 sugemalimab vs placebo after cCRT or sCRT in pts with unresectable stage III NSCLC: final PFS analysis of a phase 3 study. J Thorac Oncol.

[58] Girard N, Bar J, Garrido P, Garassino MC, McDonald F, Mornex F, Filippi AR, Smit H, Peters S, Field JK, Christoph DC, Sibille A, Fietkau R, Haakensen VD, Chouaid C, Markman B, Hiltermann T, Taus A, Sawyer W, Allen A, Chander P, Licour M, Solomon B (2023). Treatment characteristics and real-world progression-free survival in patients with unresectable stage III NSCLC who received durvalumab after chemoradiotherapy: findings from the PACIFIC-R study. J Thorac Oncol.

[59] Felip E, Rosell R, Maestre JA, Rodríguez-Paniagua JM, Morán T, Astudillo J, Alonso G, Borro JM, González-Larriba JL, Torres A, Camps C, Guijarro R, Isla D, Aguiló R, Alberola V, Padilla J, Sánchez-Palencia A, Sánchez JJ, Hermosilla E, Massuti B (2010). Preoperative chemotherapy plus surgery versus surgery plus adjuvant chemotherapy versus surgery alone in early-stage non-small-cell lung cancer. J Clin Oncol.

[60] Scagliotti GV, Pastorino U, Vansteenkiste JF, Spaggiari L, Facciolo F, Orlowski TM, Maiorino L, Hetzel M, Leschinger M, Visseren-Grul C, Torri V (2012). Randomized phase III study of surgery alone or surgery plus preoperative cisplatin and gemcitabine in stages IB to IIIA non-small-cell lung cancer. J Clin Oncol.

[61] Forde PM, Chaft JE, Smith KN, Anagnostou V, Cottrell TR, Hellmann MD, Zahurak M, Yang SC, Jones DR, Broderick S, Battafarano RJ, Velez MJ, Rekhtman N, Olah Z, Naidoo J, Marrone KA, Verde F, Guo H, Zhang J, Caushi JX, Chan HY, Sidhom JW, Scharpf RB, White J, Gabrielson E, Wang H, Rosner GL, Rusch V, Wolchok JD, Merghoub T, Taube JM, Velculescu VE, Topalian SL, Brahmer JR, Pardoll DM (2018). Neoadjuvant PD-1 blockade in resectable lung cancer. N Engl J Med.

[62] A Trial of SHR-1701 With or Without Chemotherapy in Patients With Stage III NSCLC. https://clinicaltrials.gov/ct2/show/NCT04580498 2020.

[63] ctDNA Guiding Treatment After Almonertinib Induction Therapy for EGFRm+ NSCLC in the MDT Diagnostic Model (APPROACH). https://clinicaltrials.gov/ct2/show/NCT04841811 2021.

[64] Mazieres J, Drilon A, Lusque A, Mhanna L, Cortot AB, Mezquita L, Thai AA, Mascaux C, Couraud S, Veillon R, Van den Heuvel M, Neal J, Peled N, Früh M, Ng TL, Gounant V, Popat S, Diebold J, Sabari J, Zhu VW, Rothschild SI, Bironzo P, Martinez-Marti A, Curioni-Fontecedro A, Rosell R, Lattuca-Truc M, Wiesweg M, Besse B, Solomon B, Barlesi F, Schouten RD, Wakelee H, Camidge DR, Zalcman G, Novello S, Ou SI, Milia J, Gautschi O (2019). Immune checkpoint inhibitors for patients with advanced lung cancer and oncogenic driver alterations: results from the IMMUNOTARGET registry. Ann Oncol.

[65] Liu SY, Dong ZY, Wu SP, Xie Z, Yan LX, Li YF, Yan HH, Su J, Yang JJ, Zhou Q, Zhong WZ, Tu HY, Yang XN, Zhang XC, Wu YL (2018). Clinical relevance of PD-L1 expression and CD8+ T cells infiltration in patients with EGFR-mutated and ALK-rearranged lung cancer. Lung Cancer.

[66] A Global Study to Assess the Effects of Osimertinib Following Chemoradiation in Patients With Stage III Unresectable Non-small Cell Lung Cancer (LAURA) (LAURA). 2018.

[67] A Study Evaluating the Efficacy and Safety of Multiple Therapies in Cohorts of Participants With Locally Advanced, Unresectable, Stage III Non-Small Cell Lung Cancer (NSCLC). https://clinicaltrials.gov/ct2/show/NCT05170204 2021.

[68] A Global Study to Assess the Effects of Durvalumab + Domvanalimab Following Concurrent Chemoradiation in Participants With Stage III Unresectable NSCLC (PACIFIC-8). https://clinicaltrials.gov/ct2/show/NCT05211895 2022.

[69] A Global Study to Assess the Effects of Durvalumab With Oleclumab or Durvalumab With Monalizumab Following Concurrent Chemoradiation in Patients With Stage III Unresectable Non-Small Cell Lung Cancer (PACIFIC-9). https://clinicaltrials.gov/ct2/show/NCT05221840 2022.

[70] A Study of Atezolizumab and Tiragolumab Compared With Durvalumab in Participants With Locally Advanced, Unresectable Stage III Non-Small Cell Lung Cancer (NSCLC) (SKYSCRAPER-03). https://clinicaltrials.gov/ct2/show/NCT04513925 2020.

[71] Study of Pembrolizumab/Vibostolimab (MK-7684A) in Combination With Concurrent Chemoradiotherapy Followed by Pembrolizumab/Vibostolimab Versus Concurrent Chemoradiotherapy Followed by Durvalumab in Participants With Stage III Non-small Cell Lung Cancer (MK-7684A-006/KEYVIBE-006). https://clinicaltrials.gov/ct2/show/NCT05298423 2022.

[72] Study of Pembrolizumab With Concurrent Chemoradiation Therapy Followed by Pembrolizumab With or Without Olaparib in Stage III Non-Small Cell Lung Cancer (NSCLC) (MK-7339-012/KEYLYNK-012). https://clinicaltrials.gov/ct2/show/NCT04380636 2020.

[73] A Study of Nivolumab and Ipilimumab in Untreated Participants With Stage 3 Non-small Cell Lung Cancer (NSCLC) That is Unable or Not Planned to be Removed by Surgery (CheckMate73L). https://clinicaltrials.gov/ct2/show/NCT04026412 2019.

[74] Molina JR, Yang P, Cassivi SD, Schild SE, Adjei AA (2008). Non-small cell lung cancer: epidemiology, risk factors, treatment, and survivorship. Mayo Clin Proc.

[75] Melosky B, Wheatley-Price P, Juergens RA, Sacher A, Leighl NB, Tsao MS, Cheema P, Snow S, Liu G, Card PB, Chu Q (2021). The rapidly evolving landscape of novel targeted therapies in advanced non-small cell lung cancer. Lung Cancer.

[76] Reck M, Remon J, Hellmann MD (2022). First-line immunotherapy for non-small-cell lung cancer. J Clin Oncol.

[77] Wu M, Huang Q, Xie Y, Wu X, Ma H, Zhang Y, Xia Y (2022). Improvement of the anticancer efficacy of PD-1/PD-L1 blockade via combination therapy and PD-L1 regulation. J Hematol Oncol.

[78] Hellman S, Weichselbaum RR (1995). Oligometastases. J Clin Oncol.

[79] Isbell JM, Li BT, Gomez DR (2022). The emerging role of local therapy in oligometastatic non-small cell lung cancer. J Thorac Cardiovasc Surg.

[80] Katipally RR, Pitroda SP, Juloori A, Chmura SJ, Weichselbaum RR (2022). The oligometastatic spectrum in the era of improved detection and modern systemic therapy. Nat Rev Clin Oncol.

[81] Ashworth A, Rodrigues G, Boldt G, Palma D (2013). Is there an oligometastatic state in non-small cell lung cancer? A systematic review of the literature. Lung Cancer.

[82] Guckenberger M, Lievens Y, Bouma AB, Collette L, Dekker A, DeSouza NM, Dingemans AC, Fournier B, Hurkmans C, Lecouvet FE, Meattini I, Méndez RA, Ricardi U, Russell NS, Schanne DH, Scorsetti M, Tombal B, Verellen D, Verfaillie C, Ost P (2020). Characterisation and classification of oligometastatic disease: a European Society for Radiotherapy and Oncology and European Organisation for Research and Treatment of Cancer consensus recommendation. Lancet Oncol.

[83] Lievens Y, Guckenberger M, Gomez D, Hoyer M, Iyengar P, Kindts I, Méndez Romero A, Nevens D, Palma D, Park C, Ricardi U, Scorsetti M, Yu J, Woodward WA (2020). Defining oligometastatic disease from a radiation oncology perspective: an ESTRO-ASTRO consensus document. Radiother Oncol.

[84] E^2^-RADIatE: EORTC-ESTRO RADiotherapy InfrAstrucTure for Europe (E^2^-RADIatE). https://clinicaltrials.gov/ct2/show/NCT03818503?term=OligoCare&draw=2&rank=1 2019. 10.1016/j.radonc.2026.111615 42251997

[85] Gomez DR, Blumenschein GJ, Lee JJ, Hernandez M, Ye R, Camidge DR, Doebele RC, Skoulidis F, Gaspar LE, Gibbons DL, Karam JA, Kavanagh BD, Tang C, Komaki R, Louie AV, Palma DA, Tsao AS, Sepesi B, William WN, Zhang J, Shi Q, Wang XS, Swisher SG, Heymach JV (2016). Local consolidative therapy versus maintenance therapy or observation for patients with oligometastatic non-small-cell lung cancer without progression after first-line systemic therapy: a multicentre, randomised, controlled, phase 2 study. Lancet Oncol.

[86] Gomez DR, Tang C, Zhang J, Blumenschein GJ, Hernandez M, Lee JJ, Ye R, Palma DA, Louie AV, Camidge DR, Doebele RC, Skoulidis F, Gaspar LE, Welsh JW, Gibbons DL, Karam JA, Kavanagh BD, Tsao AS, Sepesi B, Swisher SG, Heymach JV (2019). Local consolidative therapy vs maintenance therapy or observation for patients with oligometastatic non-small-cell lung cancer: long-term results of a multi-institutional, phase II, randomized study. J Clin Oncol.

[87] Iyengar P, Wardak Z, Gerber DE, Tumati V, Ahn C, Hughes RS, Dowell JE, Cheedella N, Nedzi L, Westover KD, Pulipparacharuvil S, Choy H, Timmerman RD (2018). Consolidative radiotherapy for limited metastatic non–small-cell lung cancer: a phase 2 randomized clinical trial. JAMA Oncol.

[88] Wang XS, Bai YF, Verma V, Yu RL, Tian W, Ao R, Deng Y, Xia JL, Zhu XQ, Liu H, Pan HX, Yang L, He YK, Bai HS, Luo X, Guo Y, Zhou MX, Sun YM, Zhang ZC, Li SM, Cheng X, Tan BX, Han LF, Liu YY, Zhang K, Zeng FX, Jia L, Hao XB, Wang YY, Feng G, Xie K, Lu Y, Zeng M. Randomized trial of first-line tyrosine kinase inhibitor with or without radiotherapy for synchronous oligometastatic EGFR-mutated NSCLC. J Natl Cancer Inst 2022;djac015.

[89] Planchard D, Popat S, Kerr K, Novello S, Smit EF, Faivre-Finn C, Mok TS, Reck M, Van Schil PE, Hellmann MD, Peters S. Metastatic non-small cell lung cancer: ESMO Clinical Practice Guidelines for diagnosis, treatment and follow-up††FootnotesApproved by the ESMO Guidelines Committee: February 2002, last update September 2018. This publication supersedes the previously published version—Ann Oncol 2016; 27 (Suppl 5): v1–v27. Ann Oncol 2018;29:v192-v237. 10.1093/annonc/mdy275 30285222

[90] National Comprehensive Cancer Network. NCCN Guidelines: Non-Small Cell Lung Cancer. Version 8. NCCN; 2021. https://www.nccn.org/professionals/physician_gls/pdf/nscl.pdf 2021.

[91] Palma DA, Olson R, Harrow S, Gaede S, Louie AV, Haasbeek C, Mulroy L, Lock M, Rodrigues GB, Yaremko BP, Schellenberg D, Ahmad B, Senthi S, Swaminath A, Kopek N, Liu M, Moore K, Currie S, Schlijper R, Bauman GS, Laba J, Qu XM, Warner A, Senan S (2020). Stereotactic ablative radiotherapy for the comprehensive treatment of oligometastatic cancers: long-term results of the SABR-COMET phase II randomized trial. J Clin Oncol.

[92] Tsai CJ, Yang JT, Guttmann DM, Shaverdian N, Shepherd AF, Eng J, Gelblum D, Xu AJ, Namakydoust A, Iqbal A, Mann JM, Preeshagul I, Hajj C, Gillespie EF, Sugarman S, Modi S, Dang C, Drullinsky P, Yeh R, Girshman J, Das J, Zhi W, LaPlant Q, Reyngold M, Rimner A, Shin JY, Wu AJ, Ng K, Gucalp A, Sanford R, Khan AJ, Bromberg J, Seidman AD, Comen E, Traina TA, Gomez DR, Zhang Z, Robson ME, Rudin CM, Powell SN (2021). Consolidative use of radiotherapy to block (CURB) oligoprogression—interim analysis of the first randomized study of stereotactic body radiotherapy in patients with oligoprogressive metastatic cancers of the lung and breast. Int J Radiat Oncol Biol Phys.

[93] Kirsten WH, Mayer LA (1967). Morphologic responses to a murine erythroblastosis virus. J Natl Cancer Inst.

[94] Simanshu DK, Nissley DV, McCormick F (2017). RAS proteins and their regulators in human disease. Cell.

[95] Wahl S, Dai HY, Emdal EF, Berg T, Halvorsen TO, Ottestad AL, Lund-Iversen M, Brustugun OT, Førde D, Paulsen EE, Donnem T, Andersen S, Grønberg BH, Richardsen E (2021). The prognostic effect of KRAS mutations in non-small cell lung carcinoma revisited: a Norwegian multicentre study. Cancers (Basel).

[96] Dogan S, Shen R, Ang DC, Johnson ML, D'Angelo SP, Paik PK, Brzostowski EB, Riely GJ, Kris MG, Zakowski MF, Ladanyi M (2012). Molecular epidemiology of EGFR and KRAS mutations in 3,026 lung adenocarcinomas: higher susceptibility of women to smoking-related KRAS-mutant cancers. Clin Cancer Res.

[97] Liu SY, Sun H, Zhou JY, Jie GL, Xie Z, Shao Y, Zhang X, Ye JY, Chen CX, Zhang XC, Zhou Q, Yang JJ, Wu YL (2020). Clinical characteristics and prognostic value of the KRAS G12C mutation in Chinese non-small cell lung cancer patients. Biomark Res.

[98] Ostrem JM, Peters U, Sos ML, Wells JA, Shokat KM (2013). K-Ras(G12C) inhibitors allosterically control GTP affinity and effector interactions. Nature.

[99] Hong DS, Fakih MG, Strickler JH, Desai J, Durm GA, Shapiro GI, Falchook GS, Price TJ, Sacher A, Denlinger CS, Bang YJ, Dy GK, Krauss JC, Kuboki Y, Kuo JC, Coveler AL, Park K, Kim TW, Barlesi F, Munster PN, Ramalingam SS, Burns TF, Meric-Bernstam F, Henary H, Ngang J, Ngarmchamnanrith G, Kim J, Houk BE, Canon J, Lipford JR, Friberg G, Lito P, Govindan R, Li BT (2020). KRAS(G12C) inhibition with sotorasib in advanced solid tumors. N Engl J Med.

[100] Johnson ML, de Langen AJ, Waterhouse DM, Mazieres J, Dingemans AC, Mountzios G, Pless M, Wolf J, Schuler M, Lena H, Skoulidis F, Okamoto I, Kim S, Linardou H, Novello S, Chen Y, Solomon B, Obiozor C, Wang Y, Paz-Ares L (2022). LBA10 Sotorasib versus docetaxel for previously treated non-small cell lung cancer with KRAS G12C mutation: CodeBreaK 200 phase III study. Ann Oncol.

[101] Jänne PA, Riely GJ, Gadgeel SM, Heist RS, Ou SI, Pacheco JM, Johnson ML, Sabari JK, Leventakos K, Yau E, Bazhenova L, Negrao MV, Pennell NA, Zhang J, Anderes K, Der-Torossian H, Kheoh T, Velastegui K, Yan X, Christensen JG, Chao RC, Spira AI (2022). Adagrasib in non-small-cell lung cancer harboring a KRAS(G12C) mutation. N Engl J Med.

[102] Sabari JK, Velcheti V, Shimizu K, Strickland MR, Heist RS, Singh M, Nayyar N, Giobbie-Hurder A, Digumarthy SR, Gainor JF, Rajan AP, Nieblas-Bedolla E, Burns AC, Hallin J, Olson P, Christensen JG, Kurz SC, Brastianos PK, Wakimoto H (2022). Activity of adagrasib (MRTX849) in brain metastases: preclinical models and clinical data from patients with KRASG12C-mutant non-small cell lung cancer. Clin Cancer Res.

[103] Sabari JK, Spira AI, Heist RS, Janne PA, Pacheco JM, Weiss J, Gadgeel SM, Der-Torossian H, Velastegui K, Kheoh T, Christensen JG, Negrao MV (2022). Activity of adagrasib (MRTX849) in patients with KRASG12C-mutated NSCLC and active, untreated CNS metastases in the KRYSTAL-1 trial. J Clin Oncol.

[104] Reck M, Carbone DP, Garassino M, Barlesi F (2021). Targeting KRAS in non-small-cell lung cancer: recent progress and new approaches. Ann Oncol.

[105] Punekar SR, Velcheti V, Neel BG, Wong KK (2022). The current state of the art and future trends in RAS-targeted cancer therapies. Nat Rev Clin Oncol..

[106] Sun L, Hsu M, Cohen RB, Langer CJ, Mamtani R, Aggarwal C (2021). Association between KRAS variant status and outcomes with first-line immune checkpoint inhibitor-based therapy in patients with advanced non–small-cell lung cancer. JAMA Oncol.

[107] Skoulidis F, Goldberg ME, Greenawalt DM, Hellmann MD, Awad MM, Gainor JF, Schrock AB, Hartmaier RJ, Trabucco SE, Gay L, Ali SM, Elvin JA, Singal G, Ross JS, Fabrizio D, Szabo PM, Chang H, Sasson A, Srinivasan S, Kirov S, Szustakowski J, Vitazka P, Edwards R, Bufill JA, Sharma N, Ou SI, Peled N, Spigel DR, Rizvi H, Aguilar EJ, Carter BW, Erasmus J, Halpenny DF, Plodkowski AJ, Long NM, Nishino M, Denning WL, Galan-Cobo A, Hamdi H, Hirz T, Tong P, Wang J, Rodriguez-Canales J, Villalobos PA, Parra ER, Kalhor N, Sholl LM, Sauter JL, Jungbluth AA, Mino-Kenudson M, Azimi R, Elamin YY, Zhang J, Leonardi GC, Jiang F, Wong KK, Lee JJ, Papadimitrakopoulou VA, Wistuba II, Miller VA, Frampton GM, Wolchok JD, Shaw AT, Jänne PA, Stephens PJ, Rudin CM, Geese WJ, Albacker LA, Heymach JV (2018). STK11/LKB1 mutations and PD-1 inhibitor resistance in KRAS-mutant lung adenocarcinoma. Cancer Discov.

[108] Pulciani S, Santos E, Lauver AV, Long LK, Aaronson SA, Barbacid M (1982). Oncogenes in solid human tumours. Nature.

[109] Forsythe A, Zhang W, Phillip SU, Fellous M, Korei M, Keating K (2020). A systematic review and meta-analysis of neurotrophic tyrosine receptor kinase gene fusion frequencies in solid tumors. Ther Adv Med Oncol.

[110] Liu F, Wei Y, Zhang H, Jiang J, Zhang P, Chu Q (2022). NTRK fusion in non-small cell lung cancer: diagnosis, therapy, and TRK inhibitor resistance. Front Oncol.

[111] Drilon A, Tan D, Lassen UN, Leyvraz S, Liu Y, Patel JD, Rosen L, Solomon B, Norenberg R, Dima L, Brega N, Shen L, Moreno V, Kummar S, Lin JJ (2022). Efficacy and safety of larotrectinib in patients with tropomyosin receptor kinase fusion-positive lung cancers. JCO Precis Oncol.

[112] Drilon AE, Hong DS, van Tilburg CM, Doz F, Tan DSW, Kummar S, Lin JJ, McDermott RS, Zwaan CM, Norenberg R, Fellous MM, Brega N, Xu R, Laetsch TW, Shen L (2022). Long-term efficacy and safety of larotrectinib in a pooled analysis of patients with tropomyosin receptor kinase (TRK) fusion cancer. J Clin Oncol.

[113] Krzakowski MJ, Lu S, Cousin S, Smit EF, Springfeld C, Goto K, Garrido P, Chung CH, Lin JJ, Bray VJ, Pitcher B, Zeuner H, Patel S, Bordogna W, Gelderblom H (2022). Updated analysis of the efficacy and safety of entrectinib in patients (pts) with locally advanced/metastatic NTRK fusion-positive (NTRK-fp) solid tumors. J Clin Oncol.

[114] Lin JJ, Kummar S, Tan DS, Lassen UN, Leyvraz S, Liu Y, Moreno V, Patel JD, Rosen LS, Solomon BM, Norenberg R, Dima L, Brega N, Shen L, Drilon AE (2021). Long-term efficacy and safety of larotrectinib in patients with TRK fusion-positive lung cancer. J Clin Oncol.

[115] Drilon A, Paz-Ares L, Doebele RC, Farago AF, Liu SV, Chawla SP, Tosi D, Blakely CM, Krauss JC, Bazhenova L, John T, Besse B, Wolf J, Seto T, Cho BC, Rolfo C, Osborne S, Aziez A, Demetri GD (2020). 543P Entrectinib in NTRK fusion-positive NSCLC: Updated integrated analysis of STARTRK-2, STARTRK-1 and ALKA-372-001. Ann Oncol.

[116] Dziadziuszko R, Siena S, Tan DSW, Cho BC, Ahn M, Goto K, Garrido-Lopez P, Farago AF, Loong HHF, Tosi D, John T, Wolf J, Chiu C, Liu SV, Patel MR, Drilon A, Pitcher B, Simmons B, Doebele RC (2020). 1288P Efficacy of entrectinib in patients with NTRK or ROS1 fusion-positive NSCLC with CNS metastases at baseline. Ann Oncol.

[117] Harada G, Drilon A (2022). TRK inhibitor activity and resistance in TRK fusion-positive cancers in adults. Cancer Genet.

[118] Hyman D, Kummar S, Farago A, Geoerger B, Mau-Sorensen M, Taylor M, Garralda E, Nagasubramanian R, Natheson M, Song L, Capra M, Jorgensen M, Ho A, Shukla N, Smith S, Huang X, Tuch B, Ku N, Laetsch TW, Drilon A, Hong D (2019). Abstract CT127: phase I and expanded access experience of LOXO-195 (BAY 2731954), a selective next-generation TRK inhibitor (TRKi). Cancer Res.

[119] Murray BW, Rogers E, Zhai D, Deng W, Chen X, Sprengeler PA, Zhang X, Graber A, Reich SH, Stopatschinskaja S, Solomon B, Besse B, Drilon A (2021). Molecular characteristics of repotrectinib that enable potent inhibition of TRK fusion proteins and resistant mutations. Mol Cancer Ther.

[120] Stephens P, Hunter C, Bignell G, Edkins S, Davies H, Teague J, Stevens C, O'Meara S, Smith R, Parker A, Barthorpe A, Blow M, Brackenbury L, Butler A, Clarke O, Cole J, Dicks E, Dike A, Drozd A, Edwards K, Forbes S, Foster R, Gray K, Greenman C, Halliday K, Hills K, Kosmidou V, Lugg R, Menzies A, Perry J, Petty R, Raine K, Ratford L, Shepherd R, Small A, Stephens Y, Tofts C, Varian J, West S, Widaa S, Yates A, Brasseur F, Cooper CS, Flanagan AM, Knowles M, Leung SY, Louis DN, Looijenga LH, Malkowicz B, Pierotti MA, Teh B, Chenevix-Trench G, Weber BL, Yuen ST, Harris G, Goldstraw P, Nicholson AG, Futreal PA, Wooster R, Stratton MR (2004). Lung cancer: intragenic ERBB2 kinase mutations in tumours. Nature.

[121] Pillai RN, Behera M, Berry LD, Rossi MR, Kris MG, Johnson BE, Bunn PA, Ramalingam SS, Khuri FR (2017). HER2 mutations in lung adenocarcinomas: a report from the lung cancer mutation consortium. Cancer-Am Cancer Soc.

[122] Arcila ME, Chaft JE, Nafa K, Roy-Chowdhuri S, Lau C, Zaidinski M, Paik PK, Zakowski MF, Kris MG, Ladanyi M (2012). Prevalence, clinicopathologic associations, and molecular spectrum of ERBB2 (HER2) tyrosine kinase mutations in lung adenocarcinomas. Clin Cancer Res.

[123] Baselga J (2010). Treatment of HER2-overexpressing breast cancer. Ann Oncol.

[124] Friedlaender A, Subbiah V, Russo A, Banna GL, Malapelle U, Rolfo C, Addeo A (2022). EGFR and HER2 exon 20 insertions in solid tumours: from biology to treatment. Nat Rev Clin Oncol.

[125] Dziadziuszko R, Smit EF, Dafni U, Wolf J, Wasąg B, Biernat W, Finn SP, Kammler R, Tsourti Z, Rabaglio M, Ruepp B, Roschitzki-Voser H, Stahel RA, Felip E, Peters S (2019). Afatinib in NSCLC With HER2 mutations: results of the prospective, open-label phase II NICHE trial of european thoracic oncology platform (ETOP). J Thorac Oncol.

[126] Li B, Gandhi L, Besse B, Jhaveri K, Mazières J, Boni V, Shapiro G, Waqar S, Viteri S, Park H, Quinn D, Stemmer S, Cortot A, Burkard M, Scaltriti M, Won H, Lalani A, Mcculloch L, Bebchuk J, Xu F, Bryce R, Meric-Bernstam F, Piha-Paul S, Solit D, Janne P (2021). FP14.15 neratinib-based combination therapy in HER2-mutant lung adenocarcinomas: findings from two international phase 2 studies. J Thorac Oncol.

[127] Kris MG, Camidge DR, Giaccone G, Hida T, Li BT, O'Connell J, Taylor I, Zhang H, Arcila ME, Goldberg Z, Jänne PA (2015). Targeting HER2 aberrations as actionable drivers in lung cancers: phase II trial of the pan-HER tyrosine kinase inhibitor dacomitinib in patients with HER2-mutant or amplified tumors. Ann Oncol.

[128] Zhou C, Li X, Wang Q, Gao G, Zhang Y, Chen J, Shu Y, Hu Y, Fan Y, Fang J, Chen G, Zhao J, He J, Wu F, Zou J, Zhu X, Lin X (2020). Pyrotinib in HER2-mutant advanced lung adenocarcinoma after platinum-based chemotherapy: a multicenter, open-label, single-arm, phase II study. J Clin Oncol.

[129] Sacher A, Le X, Cornelissen R, Shum E, Suga J, Socinski M, Molina JR, Haura E, Clarke J, Bhat G, Lebel F, Garassino MC (2021). 36MO Safety, tolerability and preliminary efficacy of poziotinib with twice daily strategy in EGFR/HER2 Exon 20 mutant non-small cell lung cancer. Ann Oncol.

[130] Mazieres J, Lafitte C, Ricordel C, Greillier L, Negre E, Zalcman G, Domblides C, Madelaine J, Bennouna J, Mascaux C, Moro-Sibilot D, Pinquie F, Cortot AB, Otto J, Cadranel J, Langlais A, Morin F, Westeel V, Besse B (2022). Combination of trastuzumab, pertuzumab, and docetaxel in patients with advanced non-small-cell lung cancer harboring HER2 mutations: results from the IFCT-1703 R2D2 trial. J Clin Oncol.

[131] Li BT, Shen R, Buonocore D, Olah ZT, Ni A, Ginsberg MS, Ulaner GA, Offin M, Feldman D, Hembrough T, Cecchi F, Schwartz S, Pavlakis N, Clarke S, Won HH, Brzostowski EB, Riely GJ, Solit DB, Hyman DM, Drilon A, Rudin CM, Berger MF, Baselga J, Scaltriti M, Arcila ME, Kris MG (2018). Ado-trastuzumab emtansine for patients with HER2-mutant lung cancers: results from a phase II basket trial. J Clin Oncol.

[132] Li BT, Michelini F, Misale S, Cocco E, Baldino L, Cai Y, Shifman S, Tu HY, Myers ML, Xu C, Mattar M, Khodos I, Little M, Qeriqi B, Weitsman G, Wilhem CJ, Lalani AS, Diala I, Freedman RA, Lin NU, Solit DB, Berger MF, Barber PR, Ng T, Offin M, Isbell JM, Jones DR, Yu HA, Thyparambil S, Liao WL, Bhalkikar A, Cecchi F, Hyman DM, Lewis JS, Buonocore DJ, Ho AL, Makker V, Reis-Filho JS, Razavi P, Arcila ME, Kris MG, Poirier JT, Shen R, Tsurutani J, Ulaner GA, de Stanchina E, Rosen N, Rudin CM, Scaltriti M (2020). HER2-Mediated internalization of cytotoxic agents in ERBB2 amplified or mutant lung cancers. Cancer Discov.

[133] Li BT, Smit EF, Goto Y, Nakagawa K, Udagawa H, Mazières J, Nagasaka M, Bazhenova L, Saltos AN, Felip E, Pacheco JM, Pérol M, Paz-Ares L, Saxena K, Shiga R, Cheng Y, Acharyya S, Vitazka P, Shahidi J, Planchard D, Jänne PA (2022). Trastuzumab deruxtecan in HER2-mutant non-small-cell lung cancer. N Engl J Med.

[134] Tsurutani J, Iwata H, Krop I, Jänne PA, Doi T, Takahashi S, Park H, Redfern C, Tamura K, Wise-Draper TM, Saito K, Sugihara M, Singh J, Jikoh T, Gallant G, Li BT (2020). Targeting HER2 with trastuzumab deruxtecan: a dose-expansion, phase i study in multiple advanced solid tumors. Cancer Discov.

[135] Goto K, Sang-We K, Kubo T, Goto Y, Ahn M, Planchard D, Kim D, Yang JC, Yang T, Pereira KMC, Saxena K, Shiga R, Cheng Y, Aregay M, Jänne PA (2022). LBA55 Trastuzumab deruxtecan (T-DXd) in patients (Pts) with HER2-mutant metastatic non-small cell lung cancer (NSCLC): interim results from the phase 2 DESTINY-Lung02 trial. Ann Oncol.

[136] Akinboro O, Vallejo JJ, Mishra-Kalyani PS, Larkins EA, Drezner NL, Tang S, Pazdur R, Beaver JA, Singh H (2021). Outcomes of anti-PD-(L1) therapy in combination with chemotherapy versus immunotherapy (IO) alone for first-line (1L) treatment of advanced non-small cell lung cancer (NSCLC) with PD-L1 score 1–49%: FDA pooled analysis. J Clin Oncol.

[137] Liu SY, Wu YL (2017). Ongoing clinical trials of PD-1 and PD-L1 inhibitors for lung cancer in China. J Hematol Oncol.

[138] Sezer A, Kilickap S, Gümüş M, Bondarenko I, Özgüroğlu M, Gogishvili M, Turk HM, Cicin I, Bentsion D, Gladkov O, Clingan P, Sriuranpong V, Rizvi N, Gao B, Li S, Lee S, McGuire K, Chen CI, Makharadze T, Paydas S, Nechaeva M, Seebach F, Weinreich DM, Yancopoulos GD, Gullo G, Lowy I, Rietschel P (2021). Cemiplimab monotherapy for first-line treatment of advanced non-small-cell lung cancer with PD-L1 of at least 50%: a multicentre, open-label, global, phase 3, randomised, controlled trial. Lancet.

[139] Gogishvili M, Melkadze T, Makharadze T, Giorgadze D, Dvorkin M, Penkov K, Laktionov K, Nemsadze G, Nechaeva M, Rozhkova I, Kalinka E, Gessner C, Moreno-Jaime B, Passalacqua R, Li S, McGuire K, Kaul M, Paccaly A, Quek R, Gao B, Seebach F, Weinreich DM, Yancopoulos GD, Lowy I, Gullo G, Rietschel P (2022). Cemiplimab plus chemotherapy versus chemotherapy alone in non-small cell lung cancer: a randomized, controlled, double-blind phase 3 trial. Nat Med.

[140] Wang J, Wang Z, Wu L, Li B, Cheng Y, Li X, Wang X, Han L, Wu X, Fan Y, Yu Y, Lv D, Shi J, Huang J, Zhou S, Han B, Sun G, Guo Q, Ji Y, Zhu X (2022). Final progression-free survival, interim overall survival, and biomarker analyses of CHOICE-01: a phase III study of toripalimab versus placebo in combination with first-line chemotherapy for advanced NSCLC without EGFR/ALK mutations. J Clin Oncol.

[141] Zhou C, Wang Z, Sun Y, Cao L, Ma Z, Wu R, Yu Y, Yao W, Chang J, Chen J, Zhuang W, Cui J, Chen X, Lu Y, Shen H, Wang J, Li P, Qin M, Lu D, Yang J (2022). Sugemalimab versus placebo, in combination with platinum-based chemotherapy, as first-line treatment of metastatic non-small-cell lung cancer (GEMSTONE-302): interim and final analyses of a double-blind, randomised, phase 3 clinical trial. Lancet Oncol.

[142] Cho BC, Abreu DR, Hussein M, Cobo M, Patel AJ, Secen N, Lee KH, Massuti B, Hiret S, Yang J, Barlesi F, Lee DH, Ares LP, Hsieh RW, Patil NS, Twomey P, Yang X, Meng R, Johnson ML (2022). Tiragolumab plus atezolizumab versus placebo plus atezolizumab as a first-line treatment for PD-L1-selected non-small-cell lung cancer (CITYSCAPE): primary and follow-up analyses of a randomised, double-blind, phase 2 study. Lancet Oncol.

[143] Johnson M, Cho BC, Luft A, Alatorre-Alexander J, Geater SL, Laktionov K, Vasiliev A, Trukhin D, Kim S, Ursol G, Hussein M, Lim F, Yang C, Araujo L, Saito H, Reinmuth N, Shi X, Poole L, Peters S, Garon E, Mok T (2021). PL02.01 Durvalumab & #xb1; Tremelimumab + Chemotherapy as first-line treatment for mNSCLC: results from the phase 3 POSEIDON study. J Thorac Oncol.

[144] Nadal E, Rodriguez-Abreu D, Massuti B, Juan-Vidal O, Huidobro Vence G, Lopez R, de Castro CJ, Estival A, Campelo RG, Sullivan I, Felip E, Blasco A, Guirado M, Vilarino N, Simo M, Sanahuja M, Hernandez A, Navarro V, Bruna J (2022). Updated analysis from the ATEZO-BRAIN trial: Atezolizumab plus carboplatin and pemetrexed in patients with advanced nonsquamous non–small cell lung cancer with untreated brain metastases. J Clin Oncol.

[145] Lu S, Wu L, Jian H, Chen Y, Wang Q, Fang J, Wang Z, Hu Y, Sun M, Han L, Miao L, Ding C, Cui J, Li B, Pan Y, Li X, Ye F, Liu A, Wang K, Cang S, Zhou H, Sun X, Ferry D, Lin Y, Wang S, Zhang W, Zhang C (2022). Sintilimab plus bevacizumab biosimilar IBI305 and chemotherapy for patients with EGFR-mutated non-squamous non-small-cell lung cancer who progressed on EGFR tyrosine-kinase inhibitor therapy (ORIENT-31): first interim results from a randomised, double-blind, multicentre, phase 3 trial. Lancet Oncol.

[146] Nogami N, Barlesi F, Socinski MA, Reck M, Thomas CA, Cappuzzo F, Mok T, Finley G, Aerts JG, Orlandi F, Moro-Sibilot D, Jotte RM, Stroyakovskiy D, Villaruz LC, Rodríguez-Abreu D, Wan-Teck LD, Merritt D, Coleman S, Lee A, Shankar G, Yu W, Bara I, Nishio M (2022). IMpower150 final exploratory analyses for atezolizumab plus bevacizumab and chemotherapy in key NSCLC patient subgroups with EGFR mutations or metastases in the liver or brain. J Thorac Oncol.

[147] Mok TSK EA. Nivolumab (NIVO) + chemotherapy vs chemotherapy in patients (pts) with EGFR-mutated metastatic non-small cell lung cancer (mNSCLC) with disease progression after EGFR tyrosine kinase inhibitors (TKIs) in CheckMate 722. Ann Oncol 2022;33(suppl_9):S1560–S1597. 10.1016/annonc/annonc1134 (2022).

[148] Grant MJ, Herbst RS, Goldberg SB (2021). Selecting the optimal immunotherapy regimen in driver-negative metastatic NSCLC. Nat Rev Clin Oncol.

[149] Passaro A, Brahmer J, Antonia S, Mok T, Peters S (2022). Managing resistance to immune checkpoint inhibitors in lung cancer: treatment and novel strategies. J Clin Oncol.

[150] Cortellini A, Giusti R, Filetti M, Citarella F, Adamo V, Santini D, Buti S, Nigro O, Cantini L, Di Maio M, Aerts J, Bria E, Bertolini F, Ferrara MG, Ghidini M, Grossi F, Guida A, Berardi R, Morabito A, Genova C, Mazzoni F, Antonuzzo L, Gelibter A, Marchetti P, Chiari R, Macerelli M, Rastelli F, Della GL, Gori S, Tuzi A, De Tursi M, Di Marino P, Mansueto G, Pecci F, Zoratto F, Ricciardi S, Migliorino MR, Passiglia F, Metro G, Spinelli GP, Banna GL, Friedlaender A, Addeo A, Ficorella C, Porzio G, Tiseo M, Russano M, Russo A, Pinato DJ (2022). High familial burden of cancer correlates with improved outcome from immunotherapy in patients with NSCLC independent of somatic DNA damage response gene status. J Hematol Oncol.

[151] Chen Y, Gao M, Huang Z, Yu J, Meng X (2020). SBRT combined with PD-1/PD-L1 inhibitors in NSCLC treatment: a focus on the mechanisms, advances, and future challenges. J Hematol Oncol.

